# High-mass-resolution MALDI mass spectrometry imaging reveals detailed spatial distribution of metabolites and lipids in roots of barley seedlings in response to salinity stress

**DOI:** 10.1007/s11306-018-1359-3

**Published:** 2018-04-19

**Authors:** Lenin D. Sarabia, Berin A. Boughton, Thusitha Rupasinghe, Allison M. L. van de Meene, Damien L. Callahan, Camilla B. Hill, Ute Roessner

**Affiliations:** 1 School of BioSciences, University of Melbourne, Parkville, VIC 3010 Australia; 2 Metabolomics Australia, School of BioSciences, University of Melbourne, Parkville, VIC 3010 Australia; 3 School of Life and Environmental Sciences, Centre for Chemistry and Biotechnology, Deakin University, 221 Burwood Highway, Burwood, VIC 3125 Australia; 4 School of Veterinary and Life Sciences, Murdoch University, Murdoch, WA 6150 Australia

**Keywords:** MALDI, Metabolomics, Lipids, Salinity stress, Barley, *Hordeum vulgare* L.

## Abstract

**Introduction:**

Mass spectrometry imaging (MSI) is a technology that enables the visualization of the spatial distribution of hundreds to thousands of metabolites in the same tissue section simultaneously. Roots are below-ground plant organs that anchor plants to the soil, take up water and nutrients, and sense and respond to external stresses. Physiological responses to salinity are multifaceted and have predominantly been studied using whole plant tissues that cannot resolve plant salinity responses spatially.

**Objectives:**

This study aimed to use a comprehensive approach to study the spatial distribution and profiles of metabolites, and to quantify the changes in the elemental content in young developing barley seminal roots before and after salinity stress.

**Methods:**

Here, we used a combination of liquid chromatography–mass spectrometry (LC–MS), inductively coupled plasma mass spectrometry (ICP–MS), and matrix-assisted laser desorption/ionization (MALDI–MSI) platforms to profile and analyze the spatial distribution of ions, metabolites and lipids across three anatomically different barley root zones before and after a short-term salinity stress (150 mM NaCl).

**Results:**

We localized, visualized and discriminated compounds in fine detail along longitudinal root sections and compared ion, metabolite, and lipid composition before and after salt stress. Large changes in the phosphatidylcholine (PC) profiles were observed as a response to salt stress with PC 34:n showing an overall reduction in salt treated roots. ICP–MS analysis quantified changes in the elemental content of roots with increases of Na^+^ and decreases of K^+^ content.

**Conclusion:**

Our results established the suitability of combining three mass spectrometry platforms to analyze and map ionic and metabolic responses to salinity stress in plant roots and to elucidate tolerance mechanisms in response to abiotic stress, such as salinity stress.

**Electronic supplementary material:**

The online version of this article (10.1007/s11306-018-1359-3) contains supplementary material, which is available to authorized users.

## Introduction

Salinity has a negative effect on plant growth and development, reduces productivity and often leads to death (Chen and Murata 2002; Meng et al. 2017). Plant roots are highly plastic organs and are the first to sense and respond to exogenous stresses, such as high salinity (Ouyang et al. 2007). They play a major role in water and nutrient uptake, and anchoring plants to the soil.

Specialized developmental zones exist along the longitudinal axes of roots (Figs. S1, S2). The zone of cell division (Z1) includes the root cap and the apical meristem where cells are dividing and which give rise to different cell layers following a developmental gradient (Hochholdinger et al. 2004; Ishikawa and Evans 1995). The zone of elongation (Z2) is where newly formed cells stop dividing and increase greatly in length. Finally, adjacent to Z2 there is the zone of maturation (Z3) where the cells can further differentiate into specialized cell types, such as root hairs (Bruex et al. 2012; De Smet 2012).

Plants have developed mechanisms to temporarily adjust root growth and root architecture in response to salinity (Galvan-Ampudia and Testerink 2011). For example, a metabolomics study on two varieties of barley (*Hordeum vulgare* L.) demonstrated that the specific biochemical processes that support root development, adaptation and control of metabolic pathways as a response to salt are controlled in a root-zone- and variety-specific manner (Shelden et al. 2016). Similarly, Hill et al. (2016) reported that the root-zone spatial gene expression response showed a transition from transcripts related to sugar-mediated metabolism at the zone of cell division (Z1) to transcripts involved in cell wall metabolism in the zone of elongation (Z2), and to defense response-related transcripts at the zone of maturation (Z3).

Thus, root development depends on several metabolic processes that are spatially distributed among the different tissues and zones of the root and are directly affected by abiotic stresses, such as salinity. Some of the metabolic changes that are induced during root development that involve primary metabolites (i.e. sugars, amino acids, and organic acids), lipids, and transcript expression in barley roots have been studied via molecular and genetic studies (Hill et al. 2016; Natera et al. 2016; Shelden et al. 2016; Widodo et al. 2009). Therefore, techniques that allow for elucidating the localization of metabolites in barley roots in a spatial manner, such as mass spectrometry imaging (MSI), can be used to gain further understanding into the molecular changes that occur in roots in response to exposure to salt.

MSI is a surface analysis technique that allows direct analysis of molecules in situ from the surface they are bound to or the matrix they are embedded in Gode and Volmer (2013). It allows for simultaneous detection of label-free endogenous biomolecules (small molecules, lipids, peptides) directly from thin snap-frozen slices of almost any biological sample, and the simultaneous multiple measurement of hundreds to possibly thousands of analytes in a single imaging experiment, providing rich high-density multi-dimensional data (Boughton et al. 2015; Chaurand et al. 1999; Kettling et al. 2014). Matrix assisted laser desorption ionization–mass spectrometry imaging (MALDI–MSI) is the most common and popular MSI technique in terms of high spatial resolution, sensitivity and ability to ionize a wide variety of chemical compounds (Caprioli et al. 1997; Gode and Volmer 2013; Palmer et al. 2016). The spatial resolution of MALDI–MSI has been routinely established in the range of 20–30 µm, some groups have demonstrated a spatial resolution down to a single-cell level ~ 10 µm (Korte et al. 2015) and 1.4 µm (Kompauer et al. 2017).

In this study, we have applied MALDI–MSI in combination with liquid chromatography–mass spectrometry (LC–MS) and inductively coupled plasma–mass spectrometry (ICP–MS) analyses of extracts to study the spatial distribution and profiles of metabolites in developing barley seminal roots before and after salt stress. The barley food cultivar Hindmarsh was selected due to its importance to barley production in Australia, as well as previously being reported as salt tolerant compared to other commercial barley varieties (Kamboj et al. 2014). Metabolic differences arising between plants grown under control and salt conditions were explored using the three separate analytical techniques. LC–MS lipidomic analysis of root extracts prepared from physically dissected seminal roots was used to elucidate the differences between the lipid profiles of the three main zones of the seminal root, and provided a qualitative validation of the spatially localized MALDI–MSI data. ICP–MS analysis of whole root extracts was used to distinguish between the elemental content of control and salt treated roots, and quantify the changes in the elemental content of roots after exposure to salt stress. This comprehensive approach aims to provide novel insights into the spatially resolved metabolome and lipidome that are observed across the root cap and cell division zone, the elongation zone and the maturation zone during root development after exposure to short term salt stress.

## Materials and methods

### Chemicals

Solvents were purchased from Merck Millipore (Bayswater, VIC, Australia), Chemicals including 2,5-dihydroxy benzoic acid, elemental red phosphorus and Supra pure® nitric acid (70%) and hydrochloric acid (30%) were purchased from Sigma-Aldrich (Castle Hill, NSW, Australia). Embedding and freezing supplies including cryofilm fitting tool set (2.0 cm), embedding container (1.5 cm × 2.0 cm), cryofilm fitting tool, embedding medium (SCEM), cryofilm 2C (9) (2.0 cm in width) were purchased from Section-Lab Co. Ltd. (Tokyo, Japan). Sectioning supplies including Menzel-Gläser Superfrost Ultra Plus Glass slides, optimal cutting temperature (OCT) compound and Feather® C35 tungsten microtome blades were purchased from Grale HDS (Ringwood, Australia). Lysing Matrix Tubes with 0.5 g Lysing Matrix D (1.4 mm ceramic spheres) were purchased from MP Biomedicals (Seven Hills, NSW, Australia). Elemental standards were purchased from PerkinElmer (Melbourne, VIC, Australia).

### Plant material and experimental conditions

The overall experimental workflow is illustrated in Fig. S3. Uniform barley (*Hordeum vulgare* L.) cv. Hindmarsh seeds were surface-sterilized and grown in a climate-controlled growth chamber under a cycle of 24 h dark at 17 °C under control (nutrient medium without additional NaCl) and salt-treated (nutrient medium containing 150 mM NaCl) conditions as previously described (Hill et al. 2016; Shelden et al. 2016). After 48 h development on agar plates, seminal roots were dissected, collected and immediately snap-frozen in liquid nitrogen, and then stored at − 80 °C.

### Light microscopy

For light microscopy, 48 h old seminal barley roots were harvested from plants grown under control and saline conditions. These roots were then imaged to determine morphological differences in the roots and cell types within the roots. Briefly, the roots were fixed with 2.5% glutaraldehyde in phosphate buffered saline (PBS), dehydrated in an ethanol series and embedded in LRW resin. The roots were longitudinally sectioned and stained with Toluidine Blue O (TBO) general cytoplasmic stain. Images were taken on the Leica DM6000 light microscope using the MetaMorph software. Cell sizes were measured using the FIJI package (ImageJ, NIH).

### Sample preparation and analysis for untargeted lipid profiling

Seminal roots were dissected in three main sections in the following steps: a 1.25 mm long section marked “Zone 1” (meristematic zone) was taken from the root tip. The second section (“Zone 2”) was dissected from the elongation zone up to the third section, “Zone 3” (maturation zone), which was excised at the point of visible root hair elongation up to 3/4 of the entire root. Root sections varied in length between control and saline conditions according to the following measurements: Hindmarsh (control) Z1 0–1.25 mm, Z2 1.25–3.75 mm and Z3 3.75–6.25 mm; Hindmarsh (150 mM NaCl) Z1 0–1.25 mm, Z2 1.25–3.25 mm and Z3 3.25–5.25 mm. Seminal roots from 20 to 25 individual seedlings were pooled for each biological replicate. Four biological replicates were generated for each sample in four separate experiments totaling 24 samples.

Total lipids were extracted as previously described in Natera et al. (2016). Briefly, lipid extracts were obtained from 25 mg (accurate weight was recorded) of frozen and sectioned root tissue. Root tissue was transferred into pre-chilled cryo-mill tubes with 150 µL of 100% methanol containing 0.01% BHT. The solution was homogenized for 3 × 45 s using a cryo-mill at − 10 °C and 300 µL 100% chloroform were added to the mixture. The mixture was then vortexed and incubated at 37 °C for 15 min at 750 rpm. The mixture was centrifuged at 13,000 rpm for 10 min at room temperature. The supernatant was separated from the mixture and dried in a SpeedVac without heating. The samples were then stored at − 20 °C until analysis.

Dried lipid extracts were re-suspended in 100 µL of butanol/methanol (1:1, v/v) containing 5 mM ammonium formate and 1 µL aliquots were injected onto a Poroshell 120, EC-C8, 2.1 × 150 mm (2.7 µm particle size) column (Agilent Technologies) at 15 °C using an Agilent LC 1290 (Agilent Technologies, Mulgrave, Australia). Lipids were eluted at 0.26 mL/min over 30 min with a binary gradient of ACN–water (60:40, v/v) and IPA–ACN (90:10, v/v) as described by Hu et al. (2008). MS data were acquired on a TripleTOF® 6600 (AB Sciex) equipped with an ESI source in positive and negative ion mode. The MS data presented corresponds to four pooled biological replicates for each treatment group.

### HPLC–MS data processing and analysis

Raw HPLC–MS data was visually inspected for integrity using PeakView AB Sciex Software (ver. 2.2), the raw LC-TripleTOF-MS data containing *m/z*_RT (mass to charge_retention time) and associated peak intensities were converted to ABF (analysis base file) format using the Reifycs file converter for processing using MS-DIAL 2.24 (http://prime.psc.riken.jp/Metabolomics_Software/MS-DIAL/index2.html, accessed 26 September 2016) (Tsugawa et al. 2015) and statistically analyzed using MetaboAnalyst 3.0 (http://www.metaboanalyst.ca/, accessed 25 October 2016) (Xia et al. 2015). MS-Dial export was done using the default parameters for a Lipidomics omics project except for Data collection with centroid parameters and a MS1 tolerance of 0.5 Da, Peak detection parameters with a smoothing level of five scan and minimum peak height of 2000 amplitude. The MS peak filter threshold was established after manual inspection of the raw data. Additionally, adduct ion setting was set to search for [M + H]^+^, [M + Na]^+^, [M + K]^+^ and [M + NH_4_]^+^, adducts and alignment parameters were established considering a retention time tolerance of 0.2 min and a MS1 tolerance of 0.025 Da. A matrix containing tentatively identified *m/z*_RT and associated intensity peaks was exported as a csv file.

Multivariate analysis of the data was performed using one-way ANOVA and post hoc analysis using Tukey’s honestly significant difference (HSD) test. Additional statistical analysis (Student’s t-tests) comparing each root zone and with or without salt-treatment was also performed using MetaboAnalyst to generate a list of features (*m/z*_RT) that had a false discovery rate (FDR) corrected p value (p < 0.01) fold change of two or more in peak intensity relative to the respective controls.

### Sample preparation and analysis for mass spectrometry imaging

Seminal roots were prepared according to the Kawamoto method with slight modifications (Kawamoto 2003). Briefly, 15 mm long root sections measured from the root tip were procured from seminal roots, embedded into Super Cryo Embedding Medium (SCEM, Section Lab) and then frozen in a slurry mixture of dry ice/isopropanol. Frozen samples were placed into 50 mL conical centrifuge and stored at − 80 °C until cryo-sectioning.

Frozen samples were transferred to a Leica CM 1860 cryostat at − 20 °C to thermally equilibrate for 30 min. Root tissue was cryo-sectioned at 16-µm thickness, collected with cryofilm 2C (9) (Section Lab, Japan), attached to pre-chilled glass slides with electrically conducting double-sided tape (ProSciTech, Australia), and freeze-dried under vacuum at 1.0 mbar for 30 min. Dried root sections were subjected to wet matrix deposition by spraying 50 mg/mL 2,5-dihydroxy benzoic acid (DHB) in 100% acetone using a HTX Industries TM-Sprayer™ attached to a Shimadzu LC20-AD HPLC pump. Duplicate root sections from three different seedlings were chosen to cover biological variances during germination. These six samples were treated as technical and biological replicates.

### MALDI MSI experiments

MSI experiments were carried out using a Bruker SolariX 7T XR hybrid ESI–MALDI–FT–ICR–MS equipped with a SmartBeam II UV laser. MSI analysis in positive ionization mode was carried out using optimized instrumental settings for the mass range 100–1500 *m/z* in broadband mode with a Time Domain for Acquisition of 2 M providing an estimated resolving power of 130,000 at 400 *m/z*. The laser was set to 50% power using the minimum spot size (an ovaloid shape with approximately 10 × 15 µm dimensions), 750 shots per sample spot at a frequency of 2 kHz were collected, MALDI smart walk with a 25 µm grid width, 10% grid increment and a smart walk pattern grid offset of one was enabled to enhance sampling of the MALDI spot providing an ablation spot of less than 30 × 30 µm. Mass spectra were acquired using Bruker Daltonics ftmsControl 2.1.0.

### MALDI MSI data processing and analysis

MSI spectra were manually inspected and a list of *m/z* values that showed spatial distribution on the root sections was created (Table S1). The *m/z* values were then identified by accurate precursor mass (< 5 ppm) search against the LIPID MAPS (Fahy et al. 2007), and Metlin (Smith et al. 2005) databases. All potential metabolites were detected as protonated [M + H]^+^, sodiated [M + Na]^+^ and potassiated [M + K]^+^ adducts, and were collapsed into a single ID for identification and analysis purposes.

MS images were generated using flexImaging 4.1 (Bruker Daltonics) with a mass window of ± 0.001 Da, and normalized to the root mean square (RMS).

### MALDI MSI unsupervised analysis with SCiLS Lab software

SCiLS Lab (SCiLS Lab 2016b, SCiLS GmbH) was used for unsupervised spatial identification of discriminative features between control and salt-treated, and salt-treated and control root sections. Discriminative *m/z* values were determined by a univariate measure known as the receiver operating characteristic (ROC) tool, which quantified how well the *m/z* values discriminated between two states (control and salt) (Rauser et al. 2010). Briefly, MALDI MSI reduced datasets (sqlite data) were first imported into SCiLS Lab (Thiele et al. 2014), converted to the SCiLS SL format and then normalized to the total ion count (TIC). Afterward, receiver operative characteristic (ROC) analysis of identified *m/z* values was performed, with an AUC (area under the ROC curve) of > 0.75 and < 0.25 being required, as an additional criterion to the Student’s *t* test p value of < 0.05, for a peak to be considered as statistically significant for control vs salt and salt vs control, respectively. The AUC values for discriminative *m/z* value are listed in Tables S2, S3 and S4. A good discrimination is established as an AUC value closer to 1 (abundant in group 1) or 0 (abundant in group 2), and a poor discrimination with an AUC value closer to 0.5 (Rauser et al. 2010).

### Sample preparation and analysis for ICP–MS

Trace elements were extracted from root tissues using acid digestion with aqua-regia (3:1 HCl:HNO_3_) method. Briefly, whole seminal roots were harvested, thoroughly rinsed with deionized water (18.2 MΩ, Milli-Q), dried in an oven at 80 °C for 24 h and ground to a fine powder using mortar and pestle. Ground tissue (10 mg) was accurately weighed and transferred to a 10 mL Falcon tube. Samples were then acid digested with 600 µL of aqua-regia at 80 °C for 1.5 h. After cooling to room temperature samples were diluted to a final volume of 10 mL with deionized water. Samples were mixed then centrifuged for 5 min at 5000 rpm prior to analysis by ICP–MS (Callahan et al. 2016). Elemental analysis was carried out using an inductively coupled plasma–mass spectrometer (ICP–MS; NexION 350X, PerkinElmer, USA) of the following elements P, S, K, Ca, Mg, Mn Co, Ni, Cu, Zn and Fe. The internal standards Sc (200 ppb) and Rh (20 ppb) in 1% aqua regia were used for correction. The internal standard was mixed prior to the source using a T-piece in a 1:1 ratio giving a final acid concentration of 2%. Two calibration curves were prepared one contained the high abundant elements P, Na, K and Ca at 500, 1000, 5000 ppb, the second contained the other trace elements (Zn, Mg, Fe, Mn and Cu) at 0.1, 1, 10, 50, 100, 500 ppb. The mass spectrometer was operated in kinetic energy discrimination mode (KED) with 50 ms dwell times, 20 sweeps, one reading and three replicates. The plasma source conditions were: nebulizer gas flow 1.02 L/min, auxiliary gas flow 1.2 L/min, plasma gas flow 15 L/min, ICP RF power 1500 W.

Data analysis was carried out using Syngistix (PerkinElmer) software. Signal responses were normalized to the rhodium internal standard and sample weights and dilution factors were also used.

### µ-X-ray fluorescence analysis

The instrument used in this study was a Bruker M4 Tornado µ-XRF spectrometer equipped with a Rh anode side window X-ray tube with a polycapillary lens offering a spot size down to 20 µm (for ×10 magnification) combined with high excitation intensity. Analysis were carried out directly on the root sections prepared for MALDI imaging which were placed on the *µ*-XRF platform under 20 mbar vacuum conditions. A summary of the instrumental conditions and operating parameters applied to analyze barley root sections in this study is given in Table S5. Spectra acquisition and 2D elemental maps evaluation were carried out using the Esprit software from Bruker.

## Results

### Experimental workflow

An overall workflow is provided in Fig. S3 and described in more detail in the “Materials and methods” section. Seminal roots were harvested from seedlings of the barley cultivar Hindmarsh 4 days after imbibition, grown under control (0 mM NaCl) and salt (150 mM NaCl) conditions. Microscopy analysis revealed detailed morphological features of the root including: the epidermis, which is the outermost cell layer of the root, the root cap and the quiescent center in the zone of cell division (Z1), the stele and the cortex of the root in both the elongation and maturation zones (Z2 and Z3), and root hairs highlighting the beginning of the maturation zone (Z3) (Figs. S1, S2). Root zones were measured and length differences were found between control and salt treated roots. For instance, root cap and cell division zone (Z1) showed similar length (1.25 mm) for control and salt treated plants. By contrast, the elongation zone (Z2) showed length differences between control and salt treated roots, measuring 2.5 and 2 mm, respectively (Fig. S4).

MALDI–MSI analysis of root sections revealed salinity stress-induced changes of the metabolome at three consecutive developmental zones in the barley seminal root: root cap and cell division zone (Z1), elongation zone (Z2), and maturation zone (Z3). It was not easy to clearly distinguish between the quiescent center and cell division zone, as observed in Fig. S2, due to differences in the sectioning plane of roots. In cases where these cells layers could not be distinguished, we will refer to them as the cortex and/or stele.

Several hundreds of spatially distributed peaks were observed using MSI and the LC–MS-based profiling platforms. Nevertheless, many low abundance lipids were not reproducibly detected across both platforms due to analytical and biological variations. Thus, we limited our discussion to the more abundant metabolites and lipids that were observed across three biological replicates, and to the lipid species that were detected by both MALDI–MSI and LC–MS platforms. For a simple presentation of the MSI data, all the peaks detected in MALDI datasets are presented in Table S1.

### Analysis of the lipidome of developing seminal roots

As several functionally different root zones exist in seminal barley roots, different metabolic processes can occur between different cell types. To be able to account for these differences, we evaluated the distribution of the lipidome among three distinct root zones (i.e., root cap and cell division (Z1), elongation (Z2) and maturation (Z3) zones). Using an established untargeted lipidomics approach we evaluated the lipidome of these three root zones before and after salt treatment. The three longest seminal roots were harvested from seedlings 2 days after salt treatment, from control and saline conditions. Each sampled root was sectioned and flash frozen at three specific positions along the developmental gradient of the root, as illustrated in Fig. S4. A total 24 samples corresponding to 4 biological replicates that represent 3 root zones sections grown under salt and control conditions were analyzed using LC–MS.

Tissue wounding has been reported to lead to the activation of phospholipase D resulting in the formation of PA in castor bean leaves changing the levels of endogenous PAs (Ryu and Wang 1996). Caution has been considered during root tissue harvest to ensure that the time elapsed between harvest and freezing was kept to a necessary minimum (~ 1.5 min) to prevent unwanted lipid degradation and dramatic changes in the lipid profiles of the samples. Hence, the 24 samples have been exposed to the same experimental conditions, with their drawbacks and limitations, to ensure that a reasonable comparison of lipid profiles could be established between treatments.

This analysis revealed the relative abundances and tentative identities of 577 *m/z* values that included [M + H]^+^, [M + Na]^+^, [M + K]^+^ and [M + NH_4_]^+^ adducts. The tentative lipid identities were established using accurate mass precursor ion search (< 5 ppm) against the LIPID MAPS database (Fahy et al. 2007). Tentatively annotated lipid species with several ion adducts were collapsed into a single ID and a total of 389 tentatively identified lipid species are summarized in Table S6. The tentatively annotated lipids have been grouped together into five major lipid classes: fatty acyls (FA), glycerophospholipids (GP), glycerolipids (GL), prenol lipids (PR), polyketides (PK), sphingolipids (SL) and sterol lipids (ST), which represent 4.1, 50.6, 25.4, 1.3, 0.5, 13.9 and 4.1% of the total of tentatively annotated lipid species, respectively.

In the GP class, phosphatidylcholine (PC), phosphatidic acid (PA), phosphatidylethanolamine (PE) and phosphatidylglycerol (PG) were tentatively identified as the major lipid subclasses making up to 21.8, 19.8, 15.7 and 14.7% of the GP content, respectively. In the GL class, diacylglycerol (DAG), triacylglycerol (TAG) and sulfoquinovosyl diacylglycerol (SQDG) have been identified as the most abundant lipid subclasses that make up to 26.3, 35.4 and 15.2% of the total content of GL, respectively. Among the SL class, hexosylceramides (HexCer), lactosylceramide (LacCer), have been identified as the most abundant lipid subclasses making up to 18.5 and 20.4% of the SL content, respectively.

The principal component analysis (PCA) of the tentatively annotated lipid features showed a well-defined separation among samples from the different treatments (Fig. S5). The first and third principal components accounted for 66.8% of the variation. The PCA scatter-plot divided the samples into six main groups. Root zones contributed to the clear separation on component 1 (PC1), which described 60.3% of the variability, while salinity contributed to separation on PC3, which described 6.5% of the variability (Fig S5a). Salt stressed Zone 3 (maturation) clustered on the upper left quadrant, fully separated from all other salt stressed root zones of which salt stressed Zone 2 (elongation) clustered on the border between the upper left and upper right quadrants, and Zone 1 (root cap and cell division) clustered on the upper right quadrant. A similar clustering pattern was observed on the control root zones with Zone 3 (maturation) clustering on the bottom left quadrant, Zone 2 (elongation) clustering on the border between the bottom left and bottom right quadrants, and Zone 1 (root cap and cell division) clustering on the bottom right quadrant (Fig. S5b).

A heatmap representing the changes in lipid levels in different developmental root zones under different treatments provided an integrated view of the effect of a short-term salt stress on barley roots (Fig. S6). The results show a strong separation between the different developmental root zones with Zone 1 (root cap and cell division) showing a higher relative intensity of lipids. Additionally, using analysis of variance (ANOVA) and post-hoc analyses we have evaluated the lipid species that have changed significantly in abundance along the different zones (Z1, Z2 and Z3) of barley roots under control and salt conditions. Table S7 summarizes the number of differentially annotated lipids found to be treatment-specific and zone-specific (full data sets are provided in Supplementary Data Set S1).

Relatively few lipid species changed their spatial distribution from bulked tissue extractions as a response to salt treatment (150 mM NaCl) within individual root sections but many changed in relative response: in the root cap and cell division zone (Z1), 50 lipids were differentially abundant, with two times more lipids showing an increased concentration after salt. By contrast, in the elongation zone (Z2), 72 lipids were differentially abundant, with 5.5 times more lipids that have a decreased concentration after salt. The largest treatment-specific differential lipid responses were observed in the maturation zone (Z3), with many more lipid species showing a significantly increased concentration in this root zone after salinity treatment (112 lipids were differentially abundant).

The largest differences in magnitude and quantity were observed between the different root zones: 197 and 257 lipids were found to be differentially abundant between the root cap and cell division zone (Z1) and the elongation zone (Z2) under control and salt conditions, respectively. Similarly, 293 and 213 lipids were found to be differentially abundant between the root cap and cell division zone (Z1) and the maturation zone (Z3) under control and salt conditions, respectively. It was observed that salt stress has caused an increase of about 68 lipids and a decrease of 76 lipids that were found to be differentially abundant in the elongation zone (Z2) and the maturation zone (Z3) compared to the root cap and cell division zone (Z1). By contrast, between the elongation zone (Z2) and the maturation zone (Z3), 243 and 204 *m/z* values were found to be differentially abundant under control and salt conditions, respectively, with a reduction of 43 lipids that were significantly different between these two root zones because of salt stress (Table S7).

### Elemental analysis of barley roots

The elemental composition of total barley roots was analyzed to determine the effect of 48 h of salt stress (150 mM NaCl) to the content of macro- and micro-nutrients in barley roots (Na, K, Ca, Zn, Mg, Fe, Mn and Cu). Table 1 summarizes the content of macro and micronutrients of barley roots before and after salt treatment. Among the macronutrients, we found that the Na content was significantly altered (p > 0.001) after exposure to salt stress (150 mM NaCl) with a 6.7-fold increase in salt treated roots compared to control. By contrast, none of the other seven elements showed a statistically significant difference in barley roots after exposure to salt stress. Additionally, micro X-ray fluorescence (µ-XRF) revealed the distribution of Cl^−^ and K^+^ ions in barley root sections by measuring the flux of fluorescence through samples relative to the concentration. We were unable to observe Na^+^ due to instrumental limitations; however, we have used Cl^−^ as an indirect measure of the Na^+^ distribution in tissue. Figure S7 shows the spatial distribution of Cl^−^, as in indirect measure of Na^+^, and the distribution of K^+^ on barley root sections before and after salt stress.

**Table 1:** Elemental composition in total root extract of barley cv. Hindmarsh grown under control and salt-treated conditions (150 mM NaCl)

	Control	Salt
Na	2.89 ± 0.64	19.26 ± 1.96*
K	25.17 ± 1.33	20.84 ± 3.76
Mg	0.97 ± 0.05	1.09 ± 0.12
Ca	1.26 ± 0.38	1.06 ± 0.14
Mn	26.33 ± 3.37	36.48 ± 10.17
Fe	95.86 ± 29.84	105.85 ± 26.26
Ni	0.67 ± 0.16	1.56 ± 1.39
Cu	5.13 ± 0.64	17.59 ± 21.1
Zn	64.8 ± 17.5	56.51 ± 5.89

Data points represent as mean ± standard error, N = 3. Scale: Na, K, Mg and Ca in mg g/DW, Mn, Fe, Ni, Cu and Zn in µg g/DW

*Values with significant differences (p < 0.001)

### Identification of spatially distributed lipids on barley root sections

**Fig. 1:**
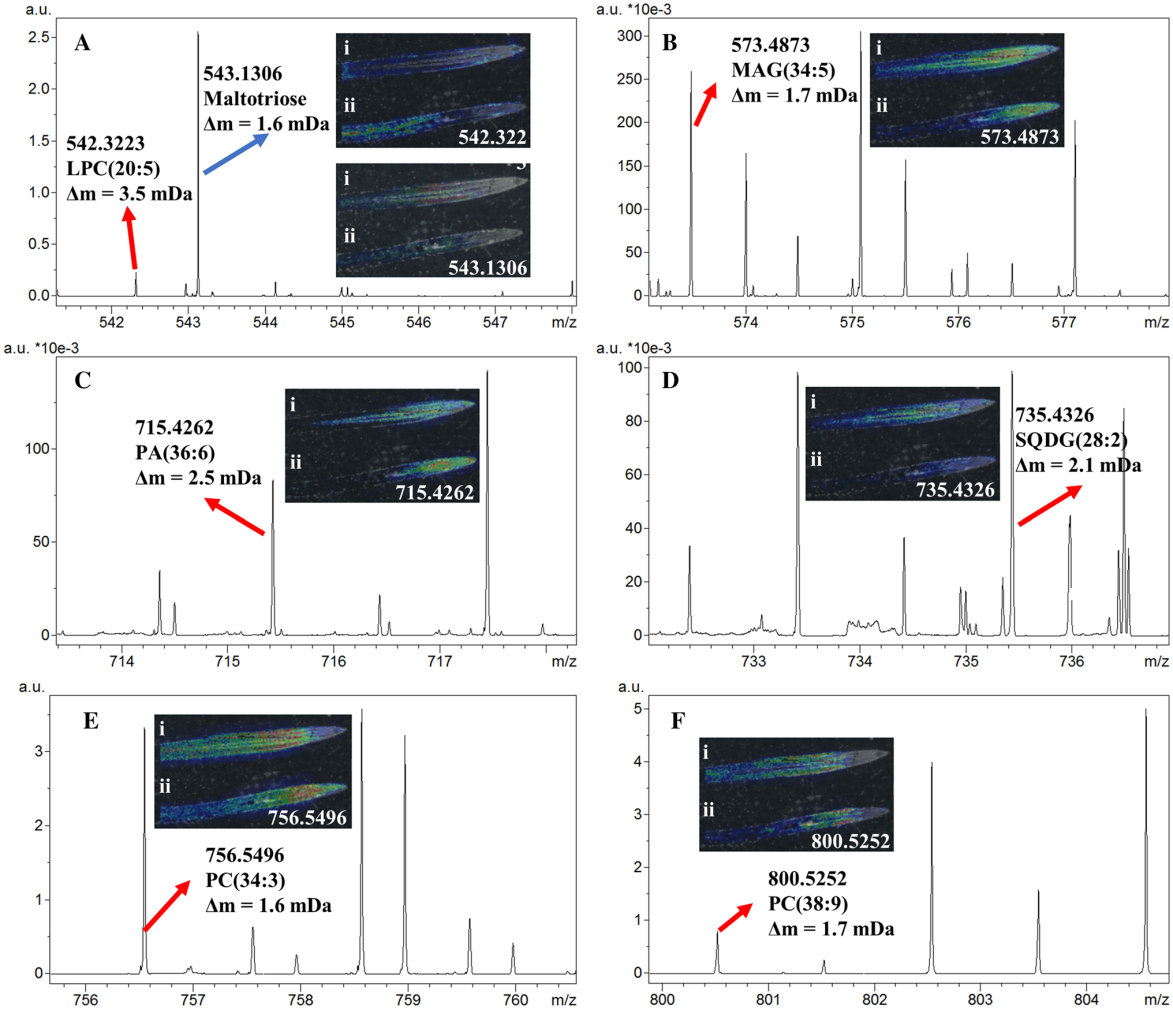
Close examination of a MALDI FT–ICR mass spectra imaging shows the peaks and the spatial distribution of tentatively identified metabolites observed in control (i) and salt treated (ii) barley root sections. *Δm m/z*-value deviation, *a.u*. arbitrary units. **a** MS-image of Maltotriose and LPC (20:5); **b** MS-image of MAG (34:5); **c** MS-image of PA (36:6); **d** MS-image of SQDG (28:2); **e** MS-image of PC (34:3) and **f** MS-image of PC (38:9)

MALDI mass spectra were manually analyzed and 277 peaks showing spatial distribution on longitudinal seminal root sections are listed in Table S1. Putative annotation using the LIPID MAPS database (accurate mass search < 5 ppm) annotated 124 tentative lipid species (Table S8) which included [M + H]^+^, [M + Na]^+^, and [M + K]^+^ ion adducts. Figure 1 shows examples of MALDI–MSI datasets and illustrates the representative lipid spectra and images that were obtained from barley longitudinal root sections. Each of the representative lipid peaks can be selected to display such distribution. The images obtained from the barley root tissue at 30 × 30 µm step size have shown differential spatial distribution across treatments and root zones. The presence and tissue distribution of these tentatively identified lipids were similar in replicate mass spectrometry imaging experiments carried out on three independent seedlings (Fig. S8). Control and salt treated images were collected from a single MALDI–MSI experiment in order to account for variability in signal intensity when comparing changes across metabolites and treatments.

**Fig. 2:**
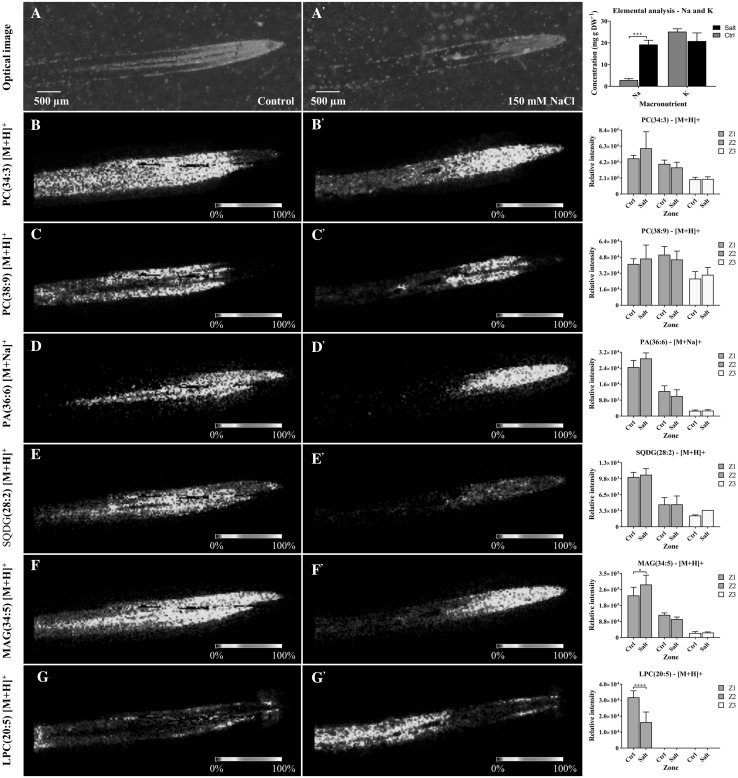
Reconstructed ion images of selected lipid species found via MALDI–MSI and confirmed by LC–MS on barley root sections under control (left panels) and 150 mM NaCl (centre panels) conditions. Right panels show a bar graph of the relative response of the lipid found on different root zones (Z1—root cap and cell division zone; Z2—zone of elongation; Z3 - zone of maturation) under control (C) and salt (S). Images were recorded with a scanning step size of 30 × 30 μm. The MS images are of PC (34:3) (*m/z* 756.5496), PC (38:9) (*m/z* 800.5252), PA (36:6) (*m/z* 715.4262), SQDG (28:2) (*m/z 735.4326), M*AG (34:*5*) (*m/z* 573.4873), and LPC (20:5) (*m/z* 542.3223). Scale bars: 500 μm. Control and salt treated images have been set to the same intensity scale and obtained from the same MALDI–MSI experiment

It is worth noting that in-source fragmentation of more complex lipid species, such as PC, during detection can lead to misidentification of fragments as other lipid species (PA, DAG, LPC, FA) (Wang 1999). However, this issue has been considered when annotating lipid species. For instance, we could establish that PC, PA, MAG and LPC species displayed different spatial distribution in tissue (Fig. 2), suggesting the detection of endogenous lipid species not the detection of other lipid fragments.

Further, tentatively annotated lipid species with several ion adducts of the same lipid species were collapsed into a single ID and the tentatively annotated lipids species were grouped together into four major lipid classes: FA, GP, GL, PK, PR, SL and ST (Table S9). They represent 10.2, 44.9, 14.2, 3.9, 6.3, 18.1 and 2.4% of the total of tentatively annotated lipid species, respectively.

Among the GP class, PC, PA and PE are the major lipid subclasses that were tentatively annotated in barley root sections and they make up to 36.8, 14 and 14% of the GP content, respectively. Regarding SL, ceramide phosphoinositol (PI-Cer) and sulfatide (SHexCer) were tentatively identified as the major lipid subclasses making up 34.7 and 30.4% of the SL content, respectively. Fatty acids contributed to 84.62% and acyl carnitines (CAR) to the 15.4% of the FA content.

PCs are differentially distributed among the root zones, depending on the molecular species, and this non-uniform distribution is affected by salt treatment. PC lipid species of the 34:n family have been found to be differentially distributed with a similar pattern across control and salt treated root sections, with differences in the *m/z*-signal intensity distribution in specific root zones. For instance, PC (34:4) was primarily found in the maturation (Z3) and elongation (Z2) zones for the control root sections, and in the elongation (Z2) and root cap and cell division (Z1) zones for the salt treated section (Fig. 2b).

PCs of the 38:n family have been found to be differentially distributed across the root sections, with a *m/z*-signal mostly observed in the elongation (Z2) and maturation (Z3) zones of both control and salt treated roots. Additionally, PC (38:9) was observed relatively unaltered in the elongation zone (Z2) of both control and salt treated barley root sections and had a relative higher intensity in the maturation zone (Z3) of the control treated root section (Fig. 2c).

PAs were found to be differentially distributed between control and salt treated roots, and across distinct root zones. PA 36:6 distribution was mostly observed in the elongation (Z2) and the root cap and cell division (Z1) zones of both control and salt treated sections. PA (36:5) distribution has been also found in the stele of the maturation zone (Z3) of a control root section, whilst it has not been observed in the maturation zone of the salt treated root section (Fig. 2d).

SQDGs distribution has been differentially observed in both control and salt treated root sections. SQDG (28:2) distribution was mostly observed across the maturation (Z3), elongation (Z2) and root cap and cell division (Z1) zones, with a slightly higher relative intensity in the elongation zone (Z2) for a control root section. By contrast, SQDG (28:2) had a significantly lower distribution in the root cap and cell division (Z1) and elongation (Z2) zones of a salt treated root section (Fig. 2e).

Monoacylglycerol (MAG) have a different spatial distribution compared to PCs, PAs or SQDGs, which were mostly found in the root cap and cell division (Z1) and elongation (Z2) zones of both control and salt treated roots. MAG (34:5) distribution also highlights the stele in the maturation zone (Z3) in the control root sections and shows a significantly lower intensity in the maturation zone (Z3) (Fig. 2f).

Lysophosphatidylcholine (LPC) lipids show a differential distribution across root zones with noticeable differences between treatments. LPC (20:5) distribution was observed across the maturation (Z3), elongation (Z2) and root cap and cell division (Z1) zones of both control and salt treated root sections highlighting several parts of the root such as the epidermis and the vascular tissue. LPC (20:5) shows an increase in the *m/z*-signal intensity in the maturation zone (Z3) of the salt treated root section (Fig. 2g).

### Identification of spatially distributed metabolites on barley root sections

In addition to the 124 tentatively assigned lipid species (Table S8), a total of 42 tentatively annotated metabolites (Tables S10 and S11) were detected and showed differential spatial distribution across treatments and root zones. Metabolite annotations were based on accurate precursor mass (< 5 ppm) search against the Metlin (Smith et al. 2005) database.

**Fig. 3:**
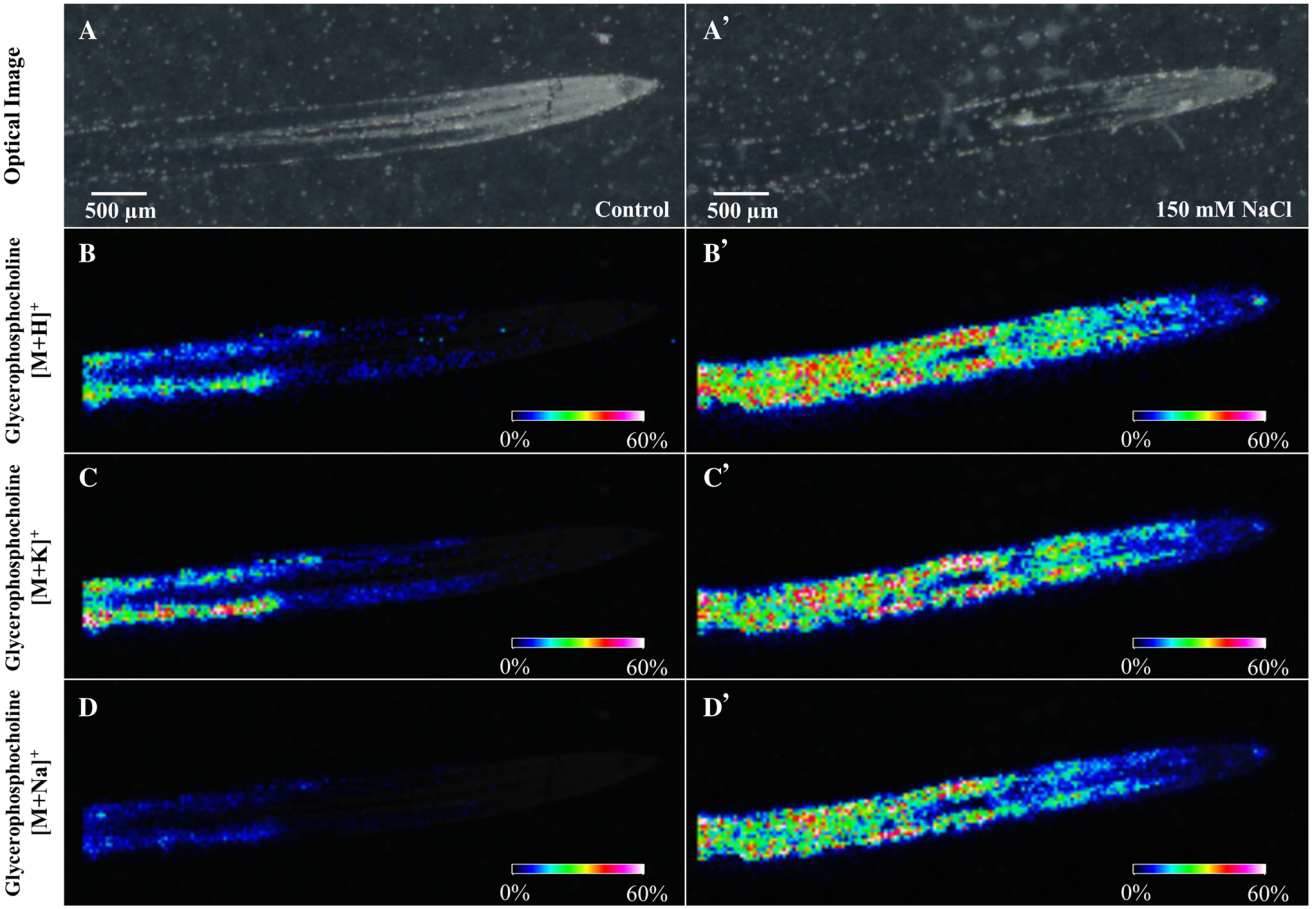
Reconstructed ion images of *Glycerophosphocholine* (GPC) *adducts* found via MALDI–MSI on barley root sections under control (left panels) and 150 mM NaCl (right panels) conditions. Images were recorded with a scanning step size of 30 × 30 μm. The MS images are of GPC—[M + H]^+^ (*m/z* 258.1117), GPC—[M + K]^+^ (*m/z* 296.0678), and GPC—[M + Na]^+^ (*m/z* 280.0928). Scale bars: 500 μm. Control and salt treated images have been set to the same intensity scale and obtained from the same MALDI–MSI experiment

Among the tentatively annotated metabolites, we have observed Glycerophosphocholine (GPC), which is a metabolite that has been proposed to be linked as a response of plants to salt stress (Aubert et al. 1996). GPC was identified as H^+^, Na^+^ and K^+^ adducts and showed significant differences in the spatial distribution between control and salt conditions (Fig. 3). For instance, GPC was mostly observed to be distributed in the cortex of the maturation zone (Z3) on a control root section. By contrast, GPC was observed more intensely distributed across the maturation (Z3), elongation (Z2) and cell division (Z1) zones of salt treated root sections. It is worth noting that endogenous GPC was also detected by LC–MS and showed an increase in the relative abundance after salt stress in both the elongation (Z2) and maturation (Z3) zones. Analysis of the fragmentation pattern of a PC standard spotted on a MALDI plate covered with DHB under the same experimental conditions did not reveal the formation of GPC. Thus, GPC endogenous levels were detected by MALDI–MSI.

**Fig. 4:**
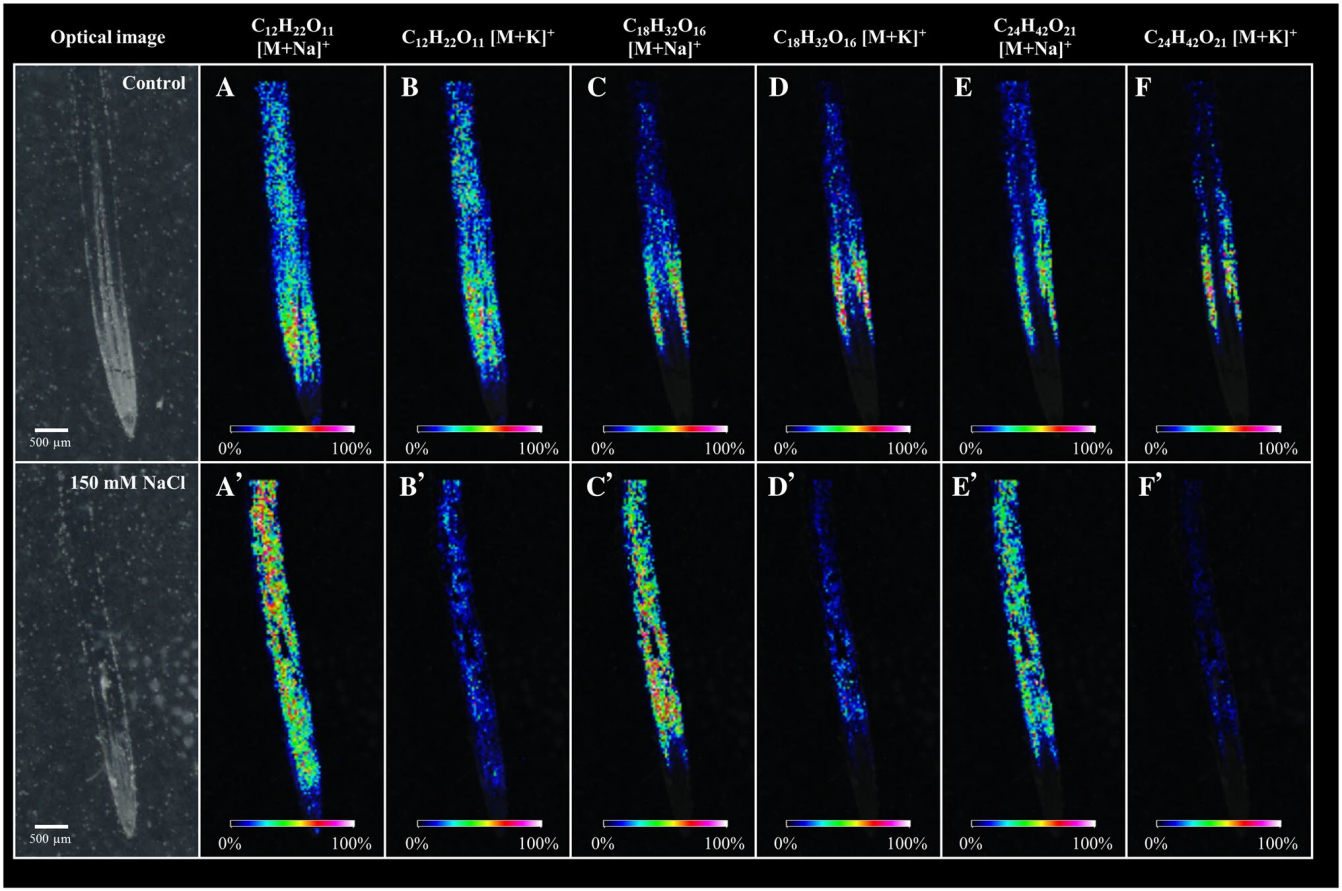
Reconstructed ion images of carbohydrates found in the barley cv. Hindmarsh root, recorded with a scanning step size of 30 × 30 μm. Optical image of a longitudinal barley root section grown in control and saline conditions. The ion images are of **a, a**′ C_12_H_22_O_11_ [M + Na]^+^ (*m/z* 365.106); **b, b**′ C_12_H_22_O_11_ [M + K]^+^ (*m/z* 381.0802); **c, c**′ C_18_H_32_O_16_ [M + Na]^+^ (*m/z* 527.1576); **d, d**′ C_18_H_32_O_16_ [M + K]^+^ (*m/z* 543.1306); **e, e**′ C_24_H_42_O_21_ [M + Na]^+^ (*m/z* 689.2068); **f, f** C_24_H_42_O_21_ [M + K]^+^ (*m/z* 705.182) Ion image are displayed using the same intensity scale (rainbow: 0–100% signal intensity). The mass accuracy was at < 5 ppm. Scale bars: 500 μm. Control and salt treated images have been set to the same intensity scale and obtained from the same MALDI–MSI experiment

Oligosaccharides with two to seven hexose units (degree of polymerization DP = 2–7) were also detected among the tentatively annotated metabolites as sodium and potassium adducts (Table S11). Figure 4 and Fig. S9 highlight the localizations of six tentatively annotated oligosaccharides in control and salt treated barley roots. We have observed that, despite minor differences, their *m/z*-signal pattern distribution is similar among these metabolites. For instance, their Na^+^ and K^+^ adducts have been observed to be distributed in the elongation (Z2) and maturation (Z3) zones of both control and salt treated root sections, except for C_12_H_22_O_11_ (DP = 2) that was also observed in the root cap and cell division zone (Z1). We have also observed that the oligosaccharides were mostly found in the elongation zone (Z2) in the control root sections for both Na^+^ and K^+^ adducts. Further, the oligosaccharides (DP = 3–7) were found mostly in the cortex of the both control and salt treated roots, whilst the disaccharide (DP = 2) was observed distributed across the whole section of both control and salt treated roots. Distribution of polysaccharides in sections of barley seeds has been previously described by Velickovic et al. (2014). However, this study describes the analysis of the distribution of polysaccharides on wheat grain after enzymatic hydrolysis of arabinoxylans and beta glucans with prior washes with ethanol and water to remove small endogenous oligosaccharides (DP = 3–6) which could interfere with the enzymatically generated beta glucans in stark contrast, we describe the spatial distribution of endogenous oligosaccharides in longitudinal sections of barley roots.

Hydroxycinnamic acid amides (HCAA) and hordatines were detected among the spatially distributed metabolites in barley roots (Table S10). Figure S10 shows the localization of one HCAA and three hordatines in control and salt treated root sections. HCAAs (coumaroylagmatine, p-coumaroylagmatine, feruloylhydroxyagmatine and feruloylagmatine) were evenly distributed across the entire longitudinal section in both control and salt treated roots, whilst hordatines were only localized in the epidermis of the root sections, as shown in Fig. S10. By contrast, HCAAs such as coumaroylagmatine and hordatines (A, B and C) were evenly distributed across the whole section of developing roots of early barley embryos (Gorzolka et al. 2016). This reveals specific localization of hordatines on the epidermis after seminal roots have fully developed and emerged.

The hordatine precursor coumaroylagmatine (CA) showed a more intense pixel distribution on the cortex of the control root section (Fig. S10b), whilst the salt treated section showed a more intense distribution of CA in the root cap and cell division zone (Z1). By contrast, hordatines with different glycosylation forms displayed similar localization patterns, as shown in Fig. S10. The glycosylated form of hordatine B (Fig. S10d, d′) revealed a more intense ion distribution mainly localized in the root cap and cell division zone than its non-glycosylated (Fig. S10c, c′) and its maltosylated forms (Fig. S10e, e′), respectively. It is also worth noting that there was a non-significant reduction in the ion intensity of hordatine B in all its glycosylated forms after salt stress.

### Validation of lipids species found by MALDI–MSI

Validation of lipid species found via MALDI–MSI was performed by finding the overlap of tentatively annotated *m/z* values that have been identified by both methods MALDI–MSI and LC–MS. There have been 389 and 124 lipid species uniquely detected in barley roots under control and salt by LC–MS and MALDI–MSI, respectively, with a total of 31 lipid species that have been detected by both methods (Table 2). Additionally, 22 of the 31 tentatively identified lipid species were further validated by LC–MS analysis in negative ion mode (Table 2). It is worth noting that LC–MS allowed us to further validate 25% of the tentatively annotated lipid species detected MALDI–MSI. Amongst the validated lipid species, fatty acids, DGDG, MAGs, LPCs, PCs, PE, PAs, and SQDGs were found, with PCs and LPCs making up to most of the validated lipid species. Figures S11, S12, S13 and S14 show MS images of validated lipid species along with a bar graph of their relative content per root zone and treatment.

**Table 2:** Tentative annotations of peaks detected in the positive ion mode MALDI–FT–ICR–MS and confirmed by LC-TripleTOF MS of the barley roots total lipid analysis

LC–MS *m/z* (Da)	Matched *m/z* (Da)	Δ*m/z* (mDa)	Name	Formula	Ion
235.1681	235.1693	1.2	1,13-Dihydroxy-herbertene*	C_15_H_23_O_2_	[M + H]^+^
361.2386	361.2374	1.2	14R,21R-diHDHA*	C_22_H_33_O_4_	[M + H]^+^
496.3391	496.3398	0.7	LPC (16:0)**	C_24_H_51_NO_7_P	[M + H]^+^
518.3227	518.3241	1.4	LPC (18:3)**	C_26_H_49_NO_7_P	[M + H]^+^
520.3389	520.3398	0.9	LPC (18:2)**	C_26_H_51_NO_7_P	[M + H]^+^
522.3537	522.3554	1.7	LPC (18:1)*	C_26_H_53_NO_7_P	[M + H]^+^
542.3206	542.3241	3.5	LPC (20:5)*	C_28_H_49_NO_7_P	[M + H]^+^
573.4861	573.4878	1.7	MAG (34:5)*	C_37_H_65_O_4_	[M + H]^+^
575.5021	575.5034	1.3	MAG (34:4)*	C_37_H_67_O_4_	[M + H]^+^
693.4474	693.4490	1.6	PA (36:6)**	C_39_H_66_O_8_P	[M + H]^+^
695.4627	695.4647	2.0	PA (36:5)**	C_39_H_68_O_8_P	[M + H]^+^
719.4602	719.4647	4.5	PA (38:7)*	C_41_H_68_O_8_P	[M + H]^+^
735.4327	735.4348	2.1	SQDG (28:2)*	C_37_H_67_O_12_S	[M + H]^+^
736.4871	736.4912	4.1	PE (36:6)**	C_41_H_71_NO_8_P	[M + H]^+^
738.5045	738.5069	2.4	PE (36:5)**	C_41_H_73_NO_8_P	[M + H]^+^
754.5359	754.5382	2.3	PC (34:4)*	C_42_H_77_NO_8_P	[M + H]^+^
756.5522	756.5538	1.6	PC (34:3)**	C_42_H_79_NO_8_P	[M + H]^+^
758.5706	758.5695	1.1	PC (34:2)**	C_42_H_81_NO_8_P	[M + H]^+^
760.5848	760.5851	0.3	PC (34:1)**	C_42_H_83_NO_8_P	[M + H]^+^
778.5393	778.5382	1.1	PC (36:6)**	C_44_H_77_NO_8_P	[M + H]^+^
780.5547	780.5538	0.9	PC (36:5)**	C_44_H_79_NO_8_P	[M + H]^+^
782.5713	782.5695	1.8	PC (36:4)**	C_44_H_81_NO_8_P	[M + H]^+^
784.5859	784.5851	0.8	PC (36:3)**	C_44_H_83_NO_8_P	[M + H]^+^
786.6005	786.6008	0.3	PC (36:2)**	C_44_H_85_NO_8_P	[M + H]^+^
800.5258	800.5225	3.3	PC (38:9)*	C_46_H_75_NO_8_P	[M + H]^+^
806.5671	806.5695	2.4	PC (38:6)*	C_46_H_81_NO_8_P	[M + H]^+^
808.5818	808.5851	3.3	PC (38:5)*	C_46_H_83_NO_8_P	[M + H]^+^
810.6006	810.6008	0.2	PC (38:4)**	C_46_H_85_NO_8_P	[M + H]^+^
857.5159	857.5175	1.6	PI (36:5)**	C_45_H_78_O_13_P	[M + H]^+^
868.6766	868.6790	2.4	PC (42:3)**	C_50_H_95_NO_8_P	[M + H]^+^
959.5686	959.5727	4.1	DGDG (38:9)*	C_53_H_83_O_15_	[M + H]^+^

Lipid tentative annotations were based on accurate precursor mass search (< 5 ppm) against the LIPID MAPS (Fahy et al. 2007) database

*Validated by LC–MS positive ion mode

**Validated by LC–MS positive and negative mode

### Analysis of discriminative metabolites and lipids observed using MALDI–MSI images

To highlight metabolites and lipid species that showed different relative abundance (pixel intensity) along the different developmental root zones (Z1, Z2 and Z3) of barley roots before and after salinity stress (150 mM NaCl), we used SCiLS Lab (Thiele et al. 2014) to test for discriminative *m/z* values for multiple interactions (treatment and zones). Control and salt treated root sections were obtained under the same experimental conditions from the same MALDI–MSI experiment. Figure S15 shows the defined root zones (Z1, Z2 and Z3) that were drawn on root sections for discriminative analysis of the tentatively annotated validated lipid species. The area under the ROC curve (AUC value) for the tentatively annotated lipids and metabolites that were discriminant between treatments are listed in Tables S2 and S3, respectively. We have chosen an AUC value of 0.75 or higher as a threshold to establish that a *m/z* value is discriminative in control treated root sections, and an AUC value of 0.25 or lower to establish that a *m/z* value is discriminative in salt treated root sections. Comparisons were carried out between equally sized sections of the three main root zones for control and salt treated barley roots.

In the comparison between control and salt treated sections (Tables S2 and S3), 2 metabolites and 21 lipids in the elongation zone (Z2) and 4 metabolites and 32 lipids in the maturation zone (Z3) were found to be discriminative (AUC > 0.75) in control root sections. By contrast, 5 metabolites and 11 lipids in the root cap and cell division zone (Z1), 7 metabolites and 9 lipids in the elongation zone (Z2) and 7 metabolites and 10 lipids in the maturation zone (Z3) were found to be discriminative (AUC < 0.25) in salt treated sections. An additional 40 unidentified peaks were also found to be discriminative between control and salt treated root sections, as listed in Table S4.

## Discussion

In this study, LC–MS and ICP–MS analyses were combined with a MALDI–MSI approach to study the untargeted lipid profile data, elemental composition profile and the spatial distribution of metabolites across differing developmental zones in seminal roots of developing barley (Hindmarsh) seedlings under control and salt conditions. These complementary techniques provided a route to analyze the metabolic and developmental differences that occur during barley root growth and finely-detailed spatial localization of metabolites. Spatial resolution is another important parameter for MALDI–MSI that directly determines the quality of the generated images of ions. A higher spatial resolution allows the visualization of cellular structures of biological samples in fine detail and with low signal to noise; however, a greater spatial resolution often results in longer acquisition times and larger file sizes (Zubair et al. 2016). Sample preparation, including sectioning and matrix application, is the first step in the MALDI–MSI process and can be a limiting factor in MALDI–MSI resolution. In our study, we were able to obtain high quality images at 30 µm laser step size which allowed us to observe the spatial distribution of a high number of lipid species, secondary metabolites and oligosaccharides from longitudinal sections of barley seminal roots. Recent MALDI–MSI analyses of roots were carried out on cross-sections with lower spatial resolution than presented in our study. For example, a 100 µm laser step size has been used to visualize saponin distribution in root cross-sections of three *Panax* species (Wang et al. 2016), a 50 µm laser spot diameter was used to analyze secondary metabolites in sections of root-nodules of Medicago (Ye et al. 2013), a 60 µm laser spot diameter was used to analyze thapsigargin in *Thapsia garganica* taproot cross sections (Andersen et al. 2017). Thus, images generated as part of this study represent an improvement of 1.6-times and 3.3-times in the quality of images than previously reported by (Wang et al. 2016) and (Ye et al. 2013), respectively.

To date, most MALDI–MSI studies of roots have been on secondary metabolites and small molecules (Li et al. 2016; Peukert et al. 2012; Wang et al. 2016; Ye et al. 2013) and the most thorough study on lipids in roots has been carried on bulked tissue (Natera et al. 2016). The protocol developed as part of this study allowed the tentative identification, curation and annotation of 124 lipid species and 42 metabolites by high-resolution MALDI–MSI (Tables S8, S10 and S11) in longitudinal barley root sections for the first time.

Lipids are key biomolecules that provide cell membrane structural integrity and energy source, play important roles in signal transduction, cytoskeletal rearrangements and membrane trafficking (Krauss and Haucke 2007). In addition to these roles, plant lipids act as stress mitigators and can reduce the severity and impact of abiotic stresses, for example by scavenging reactive oxygen species (ROS) (Mene-Saffrane et al. 2009). Alterations in the lipid profiles, metabolism and composition in response to environmental stresses including salinity were previously reported in barley: under salt stress barley cultivar Clipper roots showed more changes in phospholipids and overall lipid profiles than the Sahara cultivar (Natera et al. 2016). Further, Clipper has been reported to maintain its relative root growth under 100 mM NaCl whilst Sahara showed a significant inhibition of root elongation (Shelden et al. 2013). The barley cultivar Hindmarsh has been able to maintain its relative root growth under 150 mM NaCl salt stress and has showed significant changes in the phospholipid profiles. This is consistent with the notion that salt-resistant plants (Clipper and Hindmarsh), membrane integrity or plasma membrane integrity is more highly maintained (Chalbi et al. 2015; DuPont et al. 1988; Natera et al. 2016; Wu et al. 2005).

Signaling lipids include a diverse range of lipid species including phospholipids and sphingolipids, which are often present in low concentrations in tissues, and are quickly synthesized from pre-existing membrane lipids or intermediates. Among the detected lipids classes in this study, phospholipids made up 40.7% of the tentatively annotated species. The largest proportion of glycerophospholipids measured in roots were PCs which is in agreement with previous reports (Natera et al. 2016). We found that PC 36:5 and 36:6 (Fig. S11f, f′, g, g′) were amongst the most abundant PC species. Further, eight PC species (PC 34:1, 34:3, 36:2, 36:3, 36:4, 38:5, 38:6, 38:9) were significantly reduced after salt stress, particularly in the maturation zone (Figs. S11 and S12). As the most abundant phospholipid in extraplastidic membranes in photosynthetic eukaryotes, PC changes may have an effect in major functional and structural roles (Tasseva et al. 2004) leading to changes in membrane fluidity and permeability (Upchurch 2008). The reduction of PCs after salt stress may suggest that membrane remodeling has a central role in membrane homeostasis through the remodeling of its acyl groups in response to changing environmental and metabolic conditions (Lagace and Ridgway 2013).

The reduction of PC due to salt-stress may suggest lipid degradation by the activity of phospholipid-metabolizing enzymes phospholipase A1, phospholipase A2 and lysophospholipase leading to the formation of multiple polyunsaturated and unusual fatty acids, and GPC (Li-Beisson et al. 2013; Sahsah et al. 1998). GPC is a choline (Cho) derivative reported to accumulate in plants after physiological situations which involve membrane turnover or degradation (Aubert et al. 1996; Menegus and Fronza 1985; Roscher et al. 1998). The presence of GPC is generally interpreted as a stress-induced membrane turnover or degradation (Aubert et al. 1996). Thus, we have hypothesized that a significant increase in GPC in the zones of: cell division, elongation and maturation (Table S3, Fig. 3) that coincided with a significant reduction of PC species (as shown in bar graphs on Figs. S11 and S12) after salt stress during barley root development may help stabilize membranes, provide protein structure and function during osmotic stress (Kwon et al. 1995; Popova and Busheva 2001).

Previous studies have shown that the different barley root zones are highly specialized in their biological function as they revealed region specific responses of metabolites and transcripts in spatially resolved root metabolism (Hill et al. 2016; Shelden et al. 2016), and the findings presented in this study support this notion form a lipidomics perspective. Discriminative MALDI–MSI analysis, is an exploratory and qualitative technique and has allowed us to visually demonstrate larger lipid differences within different structural root zones than across treatments, especially in the maturation zone. It is also worth noting that when whole roots are pooled together and analyzed using a conventional lipidomics analysis, the results only represent average responses of all the changes that may be significantly variable across different root zones. Nevertheless, the use of LC–MS-based lipidomics as a complimentary technique to MALDI–MSI has allowed us to analyze a larger number of lipid species present in root tissue and to further validate the tentative ID annotations of lipids detected in a MALDI–MSI experiment.

In the current model of salt stress response and adaptation, salinity imposes two different stress factors on plants: osmotic stress due decreased water availability, which occurs within seconds to hours, and ionic stress as a result of ion uptake and solute imbalance, which occurs within days of salt exposure (Munns and Tester 2008). Accumulation of Na^+^ in plants grown under salt stress has been previously reported as detrimental to plant growth with an adverse effect on essential nutrient such as Ca^2+^, Mg^2+^ and K^+^ concentration (Munns and Tester 2008). Ionic stress was part of our experimental setup (two days of salinity stress prior to analysis), and as a result, Na^+^ competed with K^+^ as they are physicochemically similar, affecting plant enzyme function and thus impairing normal cellular processes in the root. In the ICP–MS analysis, we observed a strong significant increase in Na^+^ (6.7-fold), but no significant change in K^+^ (− 1.08-fold) content in barley roots after 48 h salt stress. As a direct consequence, the K^+^/Na^+^ ratio decreased strongly (8.71–1.08) after exposure to 48 h salt stress, consistent with a previously reported reduction of the K^+^/Na^+^ ratio in barley roots after salt stress (Shelden et al. 2013). Further, micro XRF results indirectly supported the ICP–MS results significant increase of Cl^−^ as an indirect measure of Na^+^ (Fig. S7b, b′), but no change in K^+^ levels in barley roots after salt stress (Fig. S7c, c′).

## Conclusion

We have provided a reliable and detailed methodology to study water-rich plant tissues, such as roots, using MALDI–MSI to reveal metabolite distributions and interactions across morphologically different root zones. previous attempts to examine root tissues have been limited by the fragility of the plant tissue. We have shown that a differential lipid distribution in root tissues is affected by exposure to salinity stress with major changes occurring in a zone-specific manner. The reduction of PC species and increase in GPC were observed as a response to salt stress and differed across root zones. This study shows that the availability of a MALDI–MSI protocol to study the barley root metabolic composition in combination with spatially established metabolomics and transcriptomics analysis will serve as a valuable resource for plant scientists and the wider research community. This approach will enable researchers to examine metabolic changes that occur in the roots of barley and other species in a spatial manner and aid elucidation of tolerance mechanisms in response to abiotic stress, such as salinity stress.

## Electronic supplementary material

Below is the link to the electronic supplementary material.

Supplemental Fig. S1. Brightfield longitudinal section of a 48-hour old barley cv. Hindmarsh seminal root. Images show the difference between a control (A) and a high salt (150 mM NaCl) stressed (B) root. Zone 1: root cap (RC) and cell division zone (CDZ), Zone 2: elongation zone (EZ), and Zone 3: maturation zone (MZ). Supplementary material 1 (TIF 8089 KB)

Supplemental Fig. S2. Brightfield longitudinal section of a 48-hour old barley cv. Hindmarsh seminal root. Images show the difference between a control (A) and a high salt (150 mM NaCl) stressed (B) root. Zone 1: root cap (RC) and cell division zone (CDZ), Zone 2: elongation zone (EZ), and Zone 3: maturation zone (MZ). Supplementary material 2 (TIF 3821 KB)

Supplemental Fig. S3. Overall workflow for MALDI-MSI and HPLC-MS analysis of barley roots. Supplementary material 3 (TIF 2696 KB)

Supplemental Fig. S4. Longitudinal sections of barley cv. Hindmarsh roots (control and 150 mM NaCl treated, respectively) showing the three main root regions. Z1 - root cap and meristematic zone; Z2 - zone of elongation; Z3 - zone of maturation. Supplementary material 4 (TIF 1496 KB)

Supplemental Fig. S5. Principal Component Analysis (PCA) analyses of lipids extracted from control and salt-treated Hindmarsh (H) root zones from four biological replicates as represented by different colours and symbols on the plot. PCA1 versus PC2 shows separation between root zones in: root cap and cell division zone (HZ1), elongation zone (HZ2) and maturation zone (HZ3). PCA1 versus PCA3 shows separation between treatments (control: C1, C2, C3 and C4; salt: S1, S2, S3 and S4). HZ1 (Hindmarsh – root cap and cell division zone), HZ2 (Hindmarsh – elongation zone), HZ3 (Hindmarsh – maturation zone).Supplementary material 5 (TIF 539 KB)

Supplemental S6. Clustered heatmap of the normalized metabolite relative response between root zones and treatment, and the measured lipids in Hindmarsh. Clustering of the lipids is depicted by the dendrogram at the left. Clustering of the root zones and salt treatments is depicted by the dendrogram at the top. Individual colored cells (red higher, blue lower) on the map correspond to a normalized log response value of the lipid levels, with individual lipids in rows and samples in columns. Top right-hand legend: CT - control treated; ST - salt treated. Bottom column labels: H - Hindmarsh; Z1 - root zone 1; Z2 - root zone 2; Z3 - root zone 3; C1, C2, C3, C4 control replicates 1 to 4; S1, S2, S3, S4 salt (150 mM NaCl) replicates 1 to 4. Supplementary material 6 (DOCX 5767 KB)

Supplemental Fig. S7. µ-XRF imaging of Chloride and Potassium found in the barley cv. Hindmarsh root under control and salt (150 mM NaCl) conditions. A-A’ show the digital images of the root tissue. B-B’ show unconverted fluorescence maps of Cl- as in indirect measure of Na+. C-C’ show the unconverted fluorescence maps of K+ on root sections. The root cap and cell division, elongation and maturation zones are displayed. Scanned image has a scale bar of 2000 µm with a 10x magnification. Supplementary material 7 (TIF 3923 KB)

Supplemental Fig. S8. Reconstructed ion images of a representative lipid PC 34:3 (m/z 756.5496) found on three independent experiments in the barley cv. Hindmarsh roots, recorded with a scanning step size of 30 μm by 30 μm. Replicate 1 (A – A’, B – B’); Replicate 2 (C – C’, D – D’) and Replicate 3 (E – E’, F – F’). Ion images are displayed using the same intensity scale (Rainbow: 0 – 100). The mass accuracy was at < 5 ppm. Scale bars: 500 μm. Control and salt treated images have been set to the same intensity scale and obtained from the same MALDI-MSI experiment. Supplementary material 8 (TIF 4820 KB)

Supplemental Fig. S9. Reconstructed ion images of selected oligosaccharides found in the barley cv. Hindmarsh root, recorded with a scanning step size of 30 μm by 30 μm. Optical image of a longitudinal barley root section grown in control (a) and saline (b) conditions. The ion images are of: A and A’, C_30_H_52_O_26_ [M+Na]^+^ (m/z 851.2671); B and B’, C_30_H_52_O_26_ [M+K]^+^ (m/z 867.2444); C and C’, C_36_H_62_O_31_ [M+Na]^+^ (m/z 1013.312); D and D’, C_36_H_62_O_31_ [M+Na]^+^ (m/z 1029.284); E and E’, C_42_H_72_O_36_ [M+Na]^+^ (m/z 1175.36); F and F’, C_42_H_72_O_36_ [M+K]^+^ (m/z 1191.342). Ion image are displayed using the same intensity scale (Rainbow: 0 – 100). The mass accuracy was at < 5 ppm. Scale bars: 500 μm. Control and salt treated images have been set to the same intensity scale and obtained from the same MALDI-MSI experiment. Supplementary material 9 (TIF 3604 KB)

Supplemental Fig. S10. Reconstructed ion images of hydroxycinnamic acid derivatives and hordatines found via MALDI-MSI on barley root sections under control (left panels) and 150 mM NaCl (*right* panels) conditions. Right panels show a bar graph of the relative concentration of the lipid found on different root zones (Z1 *-* root cap and cell division zone; Z2 *-* zone of elongation; Z3 *-* zone of maturation) under control (C) and salt (S). Images were recorded with a scanning step size of 30 × 30 μm. The MS images are of PC(34:4) (*m/z* 754.5343), PC(34:3) (*m/z* 756.5496), PC(34:2) (*m/z* 758.5674), PC(34:1) (*m/z* 760.5802), PC(36:6) (*m/z* 778.5359) and PC(36:5) (*m/z* 780.5515). Scale bars: 500 μm. Control and salt treated images have been set to the same intensity scale and obtained from the same MALDI-MSI experiment. Supplementary material 10 (TIF 2436 KB)

Supplemental Fig. S11. Reconstructed ion images of lipid species found via MALDI-MSI and confirmed by LC-MS on barley root sections under control (left panels) and 150 mM NaCl (middle panels) conditions. Images were recorded with a scanning step size of 30 × 30 μm. The MS images are of p-coumaroylagmatine (*m/z* 293.1608) as a representative of hydroxycinnamic amides and hordatine B as representative for hordatines: non-glycosylated hordatine B (*m/z* 581.31892), glycosylated hordatine B (*m/z* 743.3713) and maltosylated hordatine B (*m/z* 905.4242). Scale bars: 500 μm. Control and salt treated images have been set to the same intensity scale and obtained from the same MALDI-MSI experiment. Supplementary material 11 (TIF 5089 KB)

Supplemental Fig. S12. Reconstructed ion images of lipid species found via MALDI-MSI and confirmed by LC-MS on barley root sections under control (left panels) and 150 mM NaCl (middle panels) conditions. Right panels show a bar graph of the relative concentration of the lipid found on different root zones (Z1 - root cap and cell division zone; Z2 - zone of elongation; Z3 - zone of maturation) under control (C) and salt (S). Images were recorded with a scanning step size of 30 × 30 μm. The MS images are of PC(36:4) (m/z 782.57), PC(36:3) (m/z 693.4461), PC(36:2) (m/z 786.6051), PC(38:9) (m/z 800.5252), PC(38:6) (m/z 806.5703) and PC(38:5) (m/z 808.5863). Scale bars: 500 μm. Control and salt treated images have been set to the same intensity scale and obtained from the same MALDI-MSI experiment. Supplementary material 12 (TIF 4427 KB)

Supplemental Fig. S13. Reconstructed ion images of lipid species found via MALDI-MSI and confirmed by LC-MS on barley root sections under control (left panels) and 150 mM NaCl (middle panels) conditions. Right panels show a bar graph of the relative concentration of the lipid found on different root zones (Z1 - root cap and cell division zone; Z2 - zone of elongation; Z3 - zone of maturation) under control (C) and salt (S). Images were recorded with a scanning step size of 30 × 30 μm. The MS images are of MAG(34:*5*) (m/z 573.4873), MAG(34:4) (m/z 575.5033), LPC(20:5) (m/z 542.3223), LPC(18:3) (m/z 518.3257), LPC(18:2) (m/z 520.3407) *and* LPC(18:*1*) (m/z 52*2*.3*57*). Scale bars: 500 μm. Control and salt treated images have been set to the same intensity scale and obtained from the same MALDI-MSI experiment. Supplementary material 13 (TIF 4225 KB)

Supplemental Fig. S14. Reconstructed ion images of lipid species found via MALDI-MSI and confirmed by LC-MS on barley root sections under control (left panels) and 150 mM NaCl (middle panels) conditions. Right panels show a bar graph of the relative concentration of the lipid found on different root zones (Z1 - root cap and cell division zone; Z2 - zone of elongation; Z3 - zone of maturation) under control (C) and salt (S). Images were recorded with a scanning step size of 30 × 30 μm. The MS images are of PE(36:6) (m/z 736.4836), PE(36:5) (m/z 738.5002), PA(36:6) (m/z 695.4598), PA(36:6) (m/z 715.4262), PA(36:5) (m/z 693.4461) and PA(36:5) (m/z 717.44232). Scale bars: 500 μm. Control and salt treated images have been set to the same intensity scale and obtained from the same MALDI-MSI experiment.Supplementary material 14 (TIF 4029 KB)

Supplemental Fig. S15. Barley root sections divided by zone for discriminative analysis in SCiLS Lab. *CT - control; ST - salt; Z1 - root cap and cell division zone; Z2 - elongation zone; and Z3 - maturation zone*. Supplementary material 15 (TIF 798 KB)

Supplementary material 16 (DOCX 18 KB)

Supplementary material 17 (DOCX 20 KB)

Supplementary material 18 (DOCX 17 KB)

Supplementary material 19 (DOCX 18 KB)

Supplementary material 20 (DOCX 16 KB)

Supplementary material 21 (DOCX 18 KB)

Supplementary material 22 (DOCX 15 KB)

Supplementary material 23 (DOCX 34 KB)

Supplementary material 24 (DOCX 17 KB)

Supplementary material 25 (DOCX 23 KB)

Supplementary material 26 (DOCX 18 KB)

Supplemental Data Set S1. Lists of differentially annotated lipids found to be treatment-specific and one-specific. Supplementary material 27 (XLSX 63 KB)
