# Supplementary figures and images for: High-mass-resolution MALDI mass spectrometry imaging reveals detailed spatial distribution of metabolites and lipids in roots of barley seedlings in response to salinity stress
Source: Metabolomics. 2018 Apr 19;14(5):63. doi: 10.1007/s11306-018-1359-3 (PMC5907631; doi:10.1007/s11306-018-1359-3)

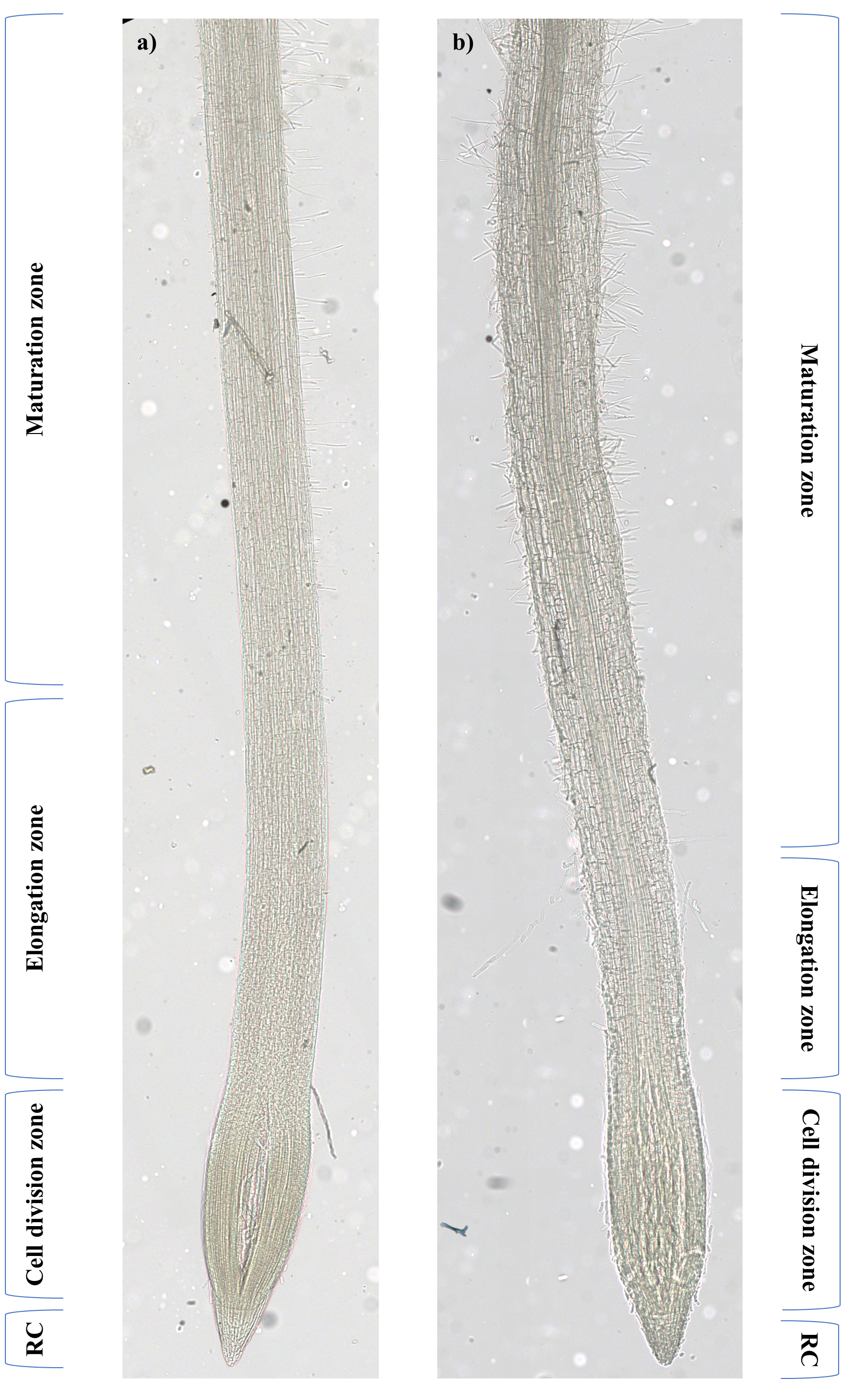

Supplement: Supplementary file 1 — Supplemental Fig. S1. Brightfield longitudinal section of a 48-hour old barley cv. Hindmarsh seminal root. Images show the difference between a control (A) and a high salt (150 mM NaCl) stressed (B) root. Zone 1: root cap (RC) and cell division zone (CDZ), Zone 2: elongation zone (EZ), and Zone 3: maturation zone (MZ). Supplementary material 1 (TIF 8089 KB) [file 11306_2018_1359_MOESM1_ESM.tif]

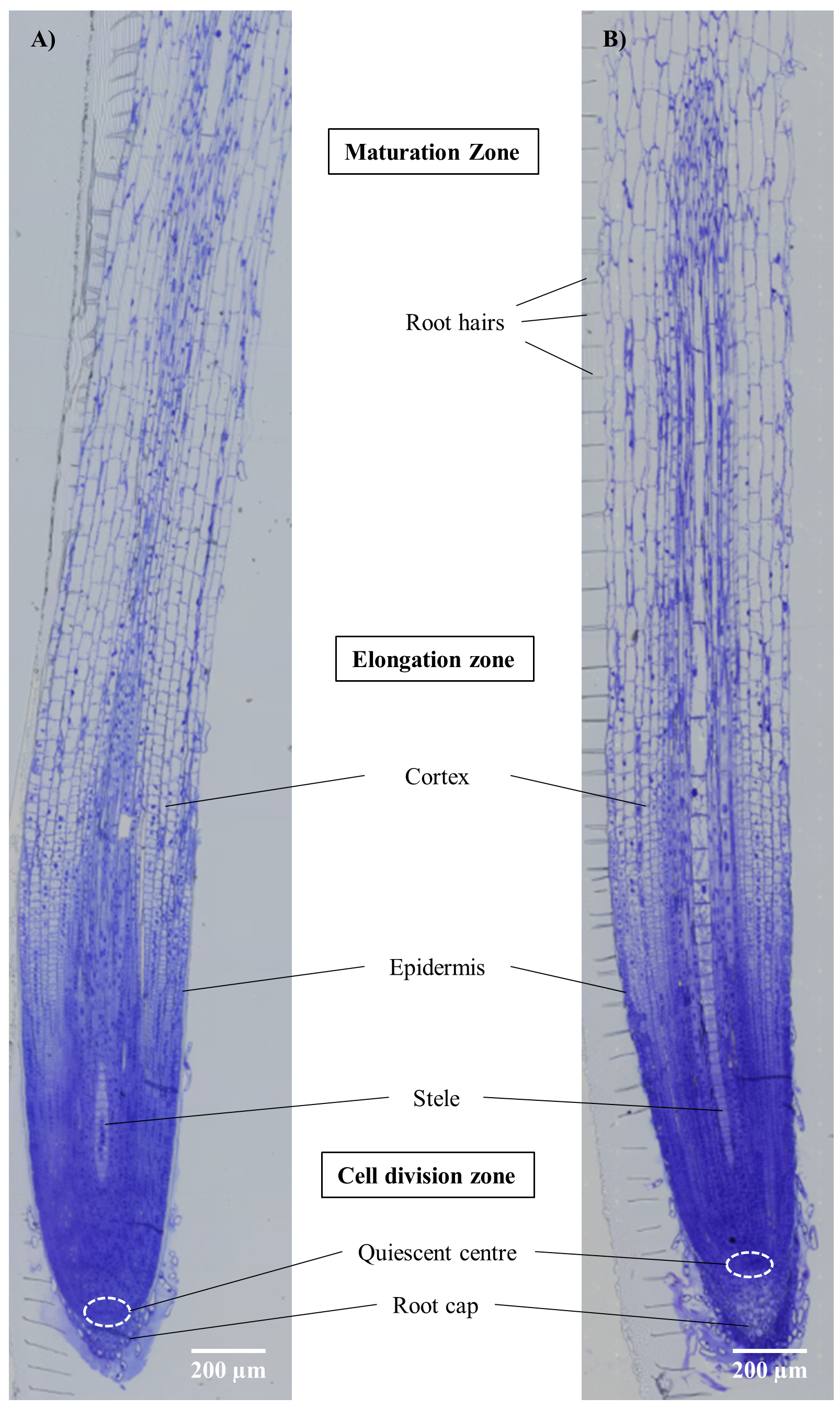

Supplement: Supplementary file 2 — Supplemental Fig. S2. Brightfield longitudinal section of a 48-hour old barley cv. Hindmarsh seminal root. Images show the difference between a control (A) and a high salt (150 mM NaCl) stressed (B) root. Zone 1: root cap (RC) and cell division zone (CDZ), Zone 2: elongation zone (EZ), and Zone 3: maturation zone (MZ). Supplementary material 2 (TIF 3821 KB) [file 11306_2018_1359_MOESM2_ESM.tif]

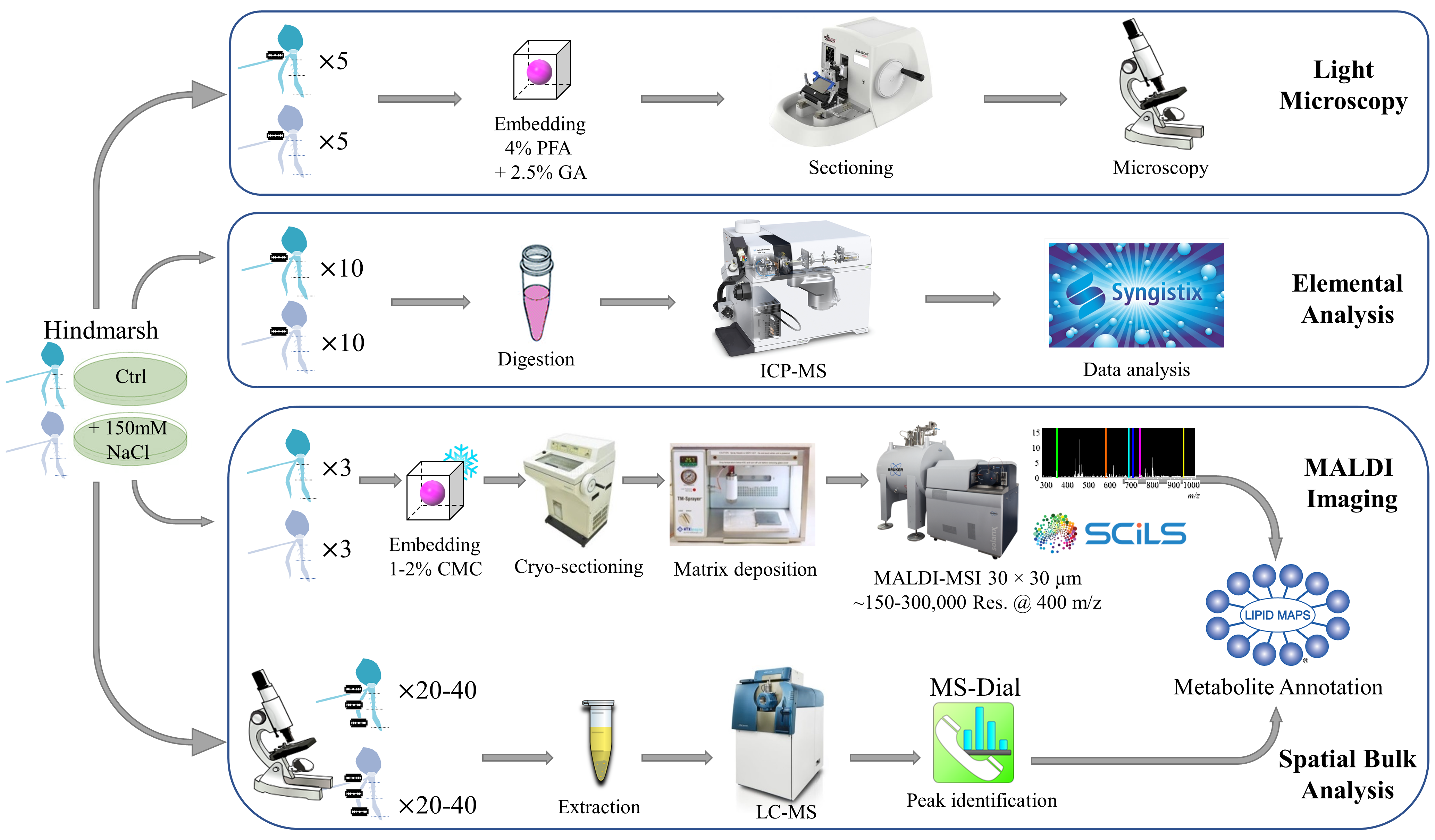

Supplement: Supplementary file 3 — Supplemental Fig. S3. Overall workflow for MALDI-MSI and HPLC-MS analysis of barley roots. Supplementary material 3 (TIF 2696 KB) [file 11306_2018_1359_MOESM3_ESM.tif]

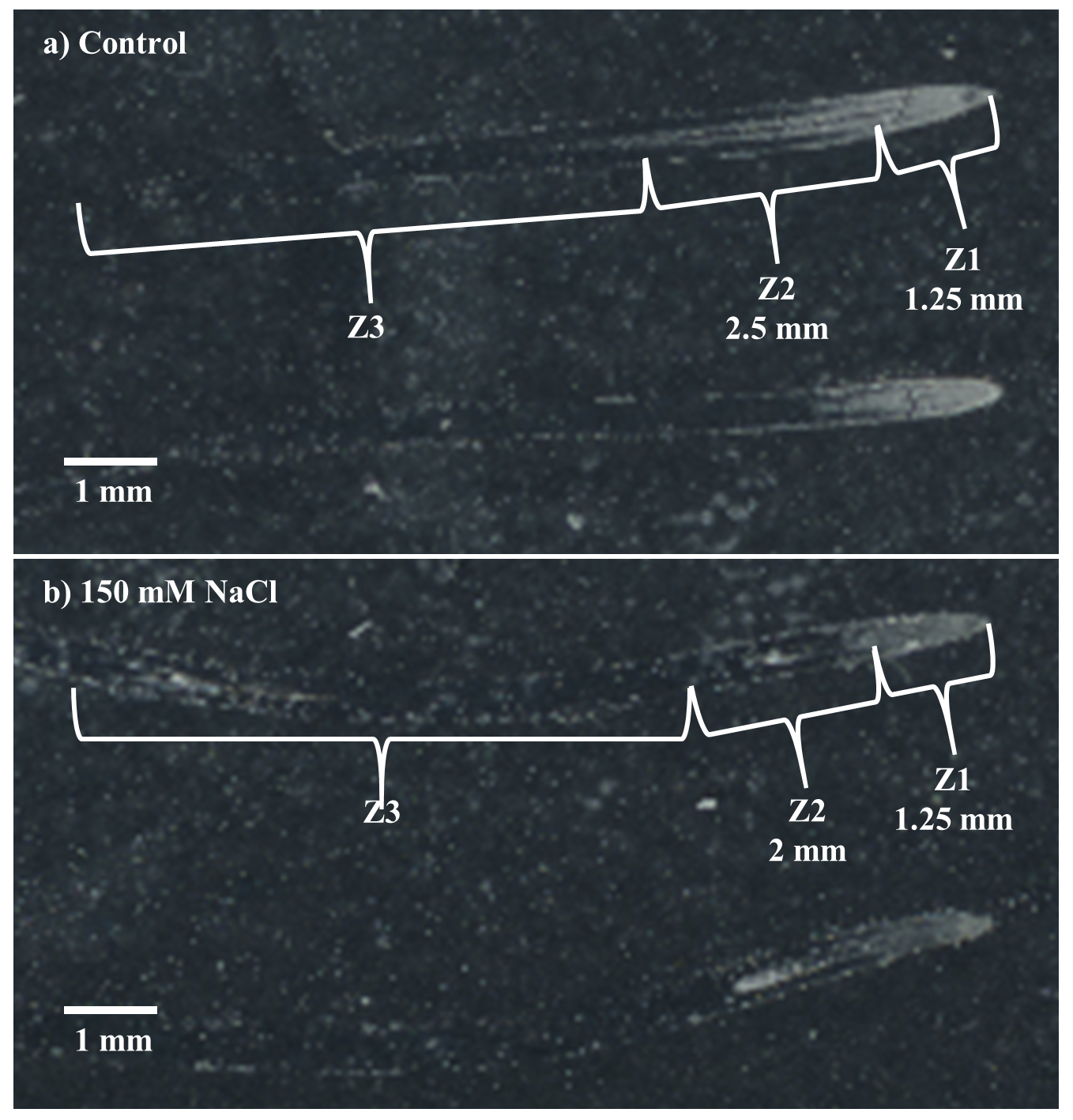

Supplement: Supplementary file 4 — Supplemental Fig. S4. Longitudinal sections of barley cv. Hindmarsh roots (control and 150 mM NaCl treated, respectively) showing the three main root regions. Z1 - root cap and meristematic zone; Z2 - zone of elongation; Z3 - zone of maturation. Supplementary material 4 (TIF 1496 KB) [file 11306_2018_1359_MOESM4_ESM.tif]

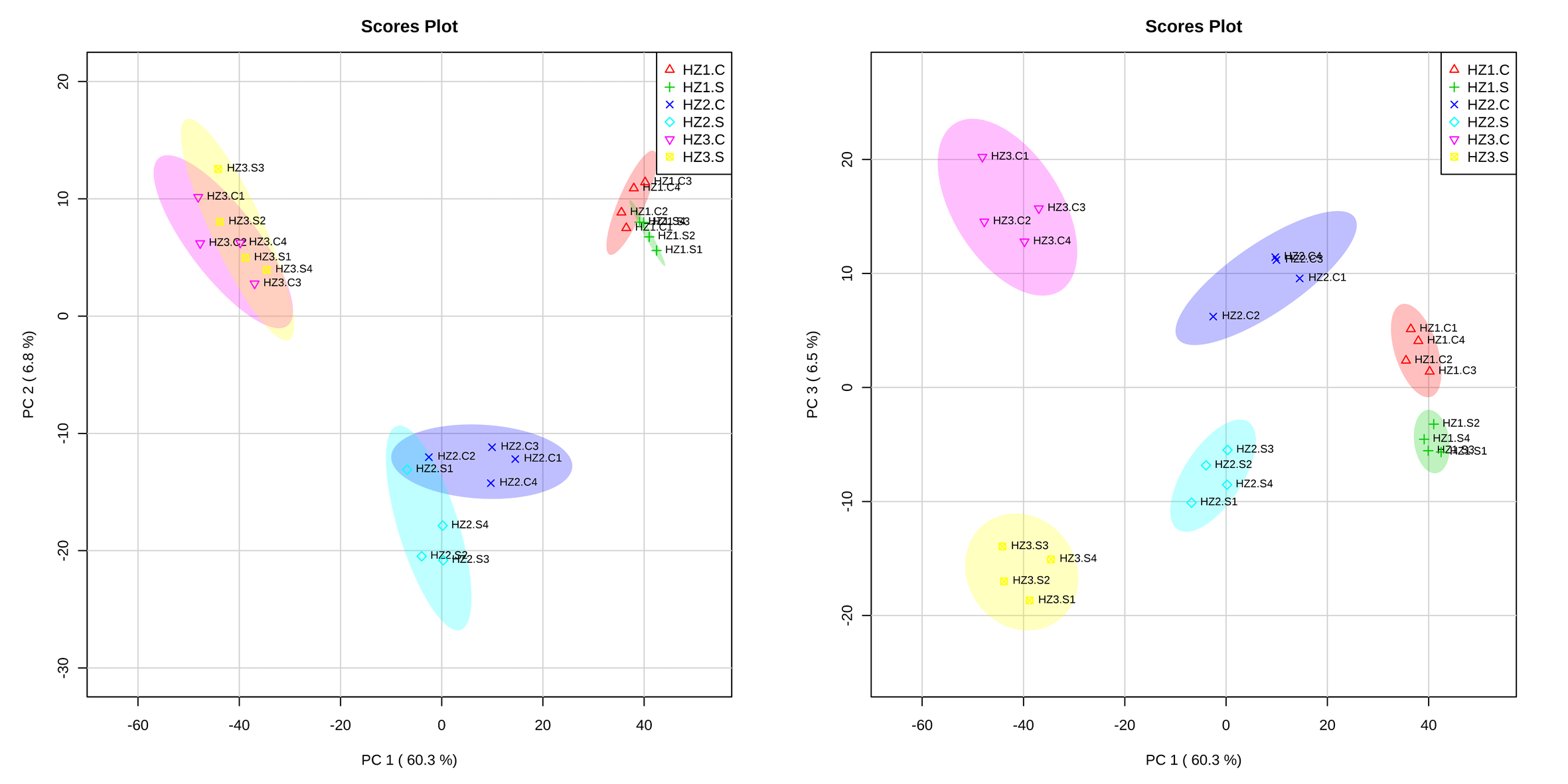

Supplement: Supplementary file 5 — Supplemental Fig. S5. Principal Component Analysis (PCA) analyses of lipids extracted from control and salt-treated Hindmarsh (H) root zones from four biological replicates as represented by different colours and symbols on the plot. PCA1 versus PC2 shows separation between root zones in: root cap and cell division zone (HZ1), elongation zone (HZ2) and maturation zone (HZ3). PCA1 versus PCA3 shows separation between treatments (control: C1, C2, C3 and C4; salt: S1, S2, S3 and S4). HZ1 (Hindmarsh – root cap and cell division zone), HZ2 (Hindmarsh – elongation zone), HZ3 (Hindmarsh – maturation zone).Supplementary material 5 (TIF 539 KB) [file 11306_2018_1359_MOESM5_ESM.tif]

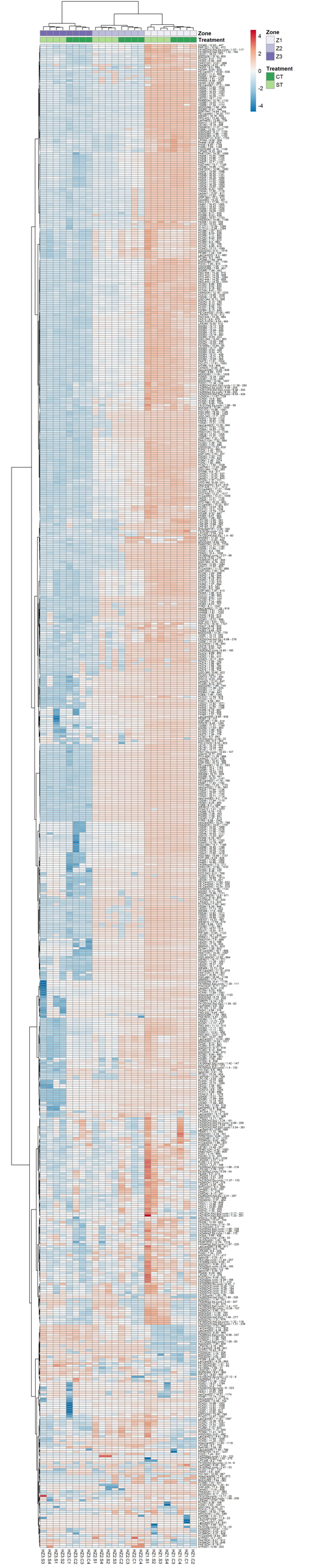

Supplement: Supplementary file 6 — Supplemental S6. Clustered heatmap of the normalized metabolite relative response between root zones and treatment, and the measured lipids in Hindmarsh. Clustering of the lipids is depicted by the dendrogram at the left. Clustering of the root zones and salt treatments is depicted by the dendrogram at the top. Individual colored cells (red higher, blue lower) on the map correspond to a normalized log response value of the lipid levels, with individual lipids in rows and samples in columns. Top right-hand legend: CT - control treated; ST - salt treated. Bottom column labels: H - Hindmarsh; Z1 - root zone 1; Z2 - root zone 2; Z3 - root zone 3; C1, C2, C3, C4 control replicates 1 to 4; S1, S2, S3, S4 salt (150 mM NaCl) replicates 1 to 4. Supplementary material 6 (DOCX 5767 KB) [file 11306_2018_1359_MOESM6_ESM.docx]

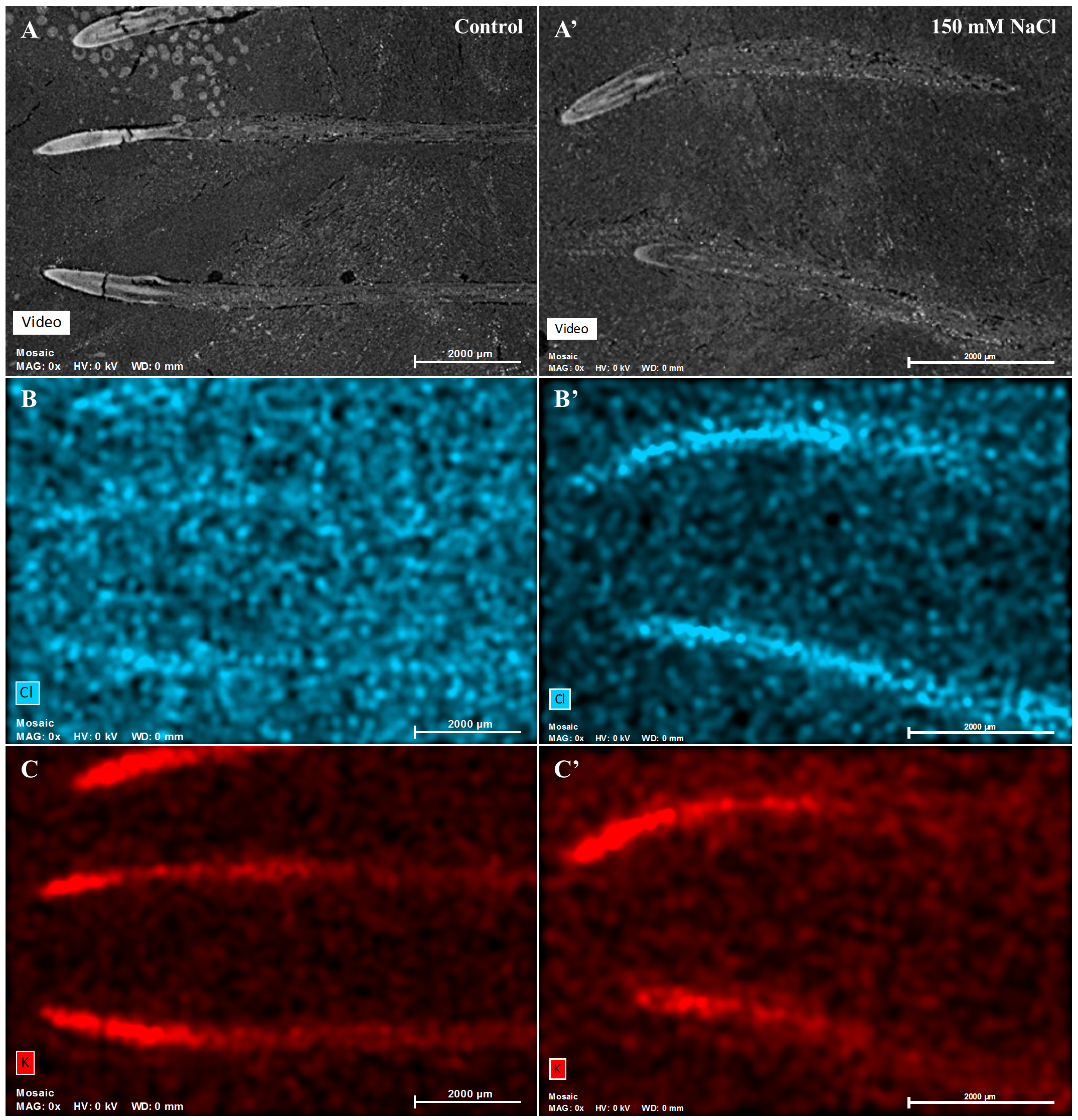

Supplement: Supplementary file 7 — Supplemental Fig. S7. µ-XRF imaging of Chloride and Potassium found in the barley cv. Hindmarsh root under control and salt (150 mM NaCl) conditions. A-A’ show the digital images of the root tissue. B-B’ show unconverted fluorescence maps of Cl- as in indirect measure of Na+. C-C’ show the unconverted fluorescence maps of K+ on root sections. The root cap and cell division, elongation and maturation zones are displayed. Scanned image has a scale bar of 2000 µm with a 10x magnification. Supplementary material 7 (TIF 3923 KB) [file 11306_2018_1359_MOESM7_ESM.tif]

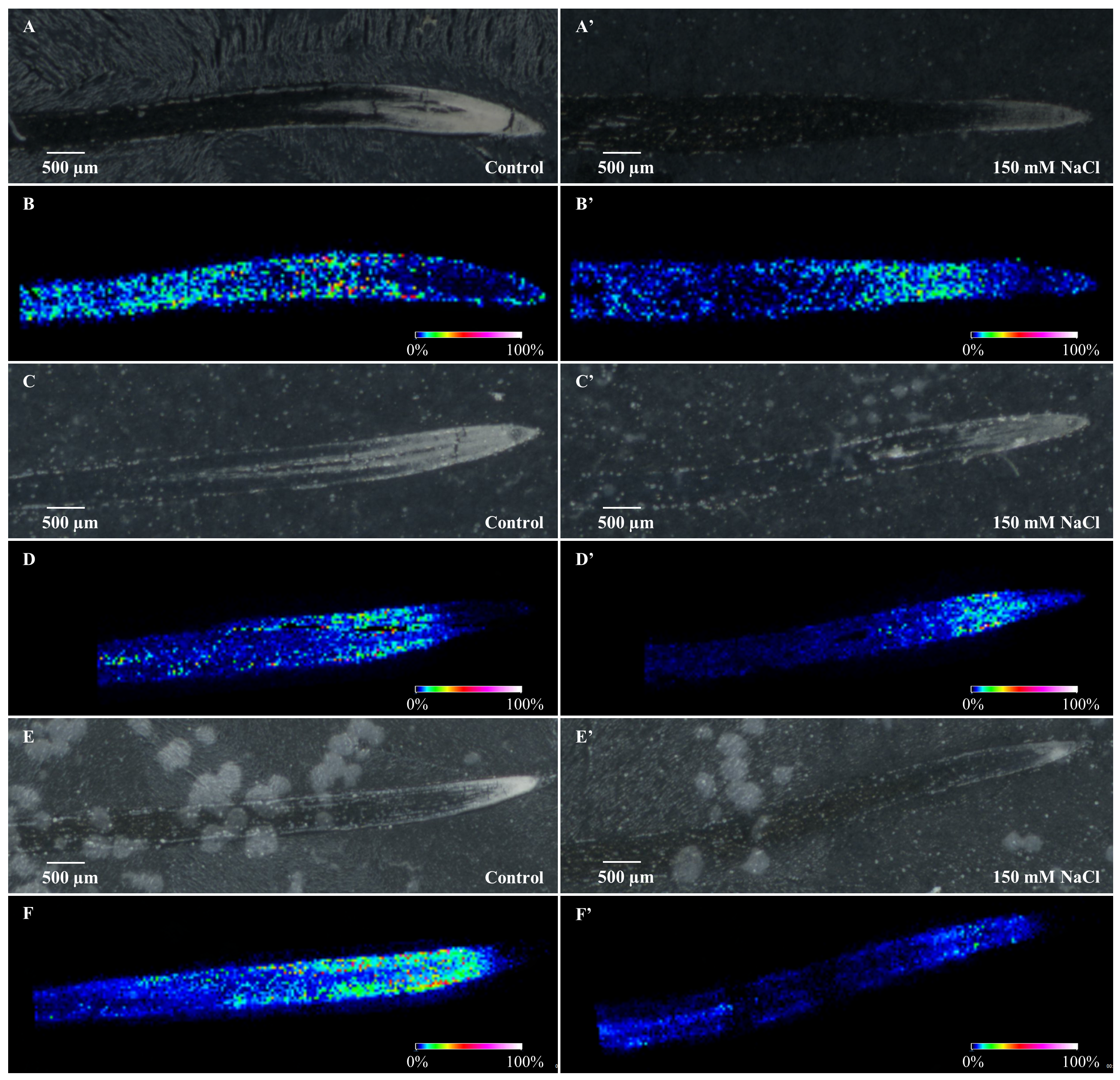

Supplement: Supplementary file 8 — Supplemental Fig. S8. Reconstructed ion images of a representative lipid PC 34:3 (m/z 756.5496) found on three independent experiments in the barley cv. Hindmarsh roots, recorded with a scanning step size of 30 μm by 30 μm. Replicate 1 (A – A’, B – B’); Replicate 2 (C – C’, D – D’) and Replicate 3 (E – E’, F – F’). Ion images are displayed using the same intensity scale (Rainbow: 0 – 100). The mass accuracy was at < 5 ppm. Scale bars: 500 μm. Control and salt treated images have been set to the same intensity scale and obtained from the same MALDI-MSI experiment. Supplementary material 8 (TIF 4820 KB) [file 11306_2018_1359_MOESM8_ESM.tif]

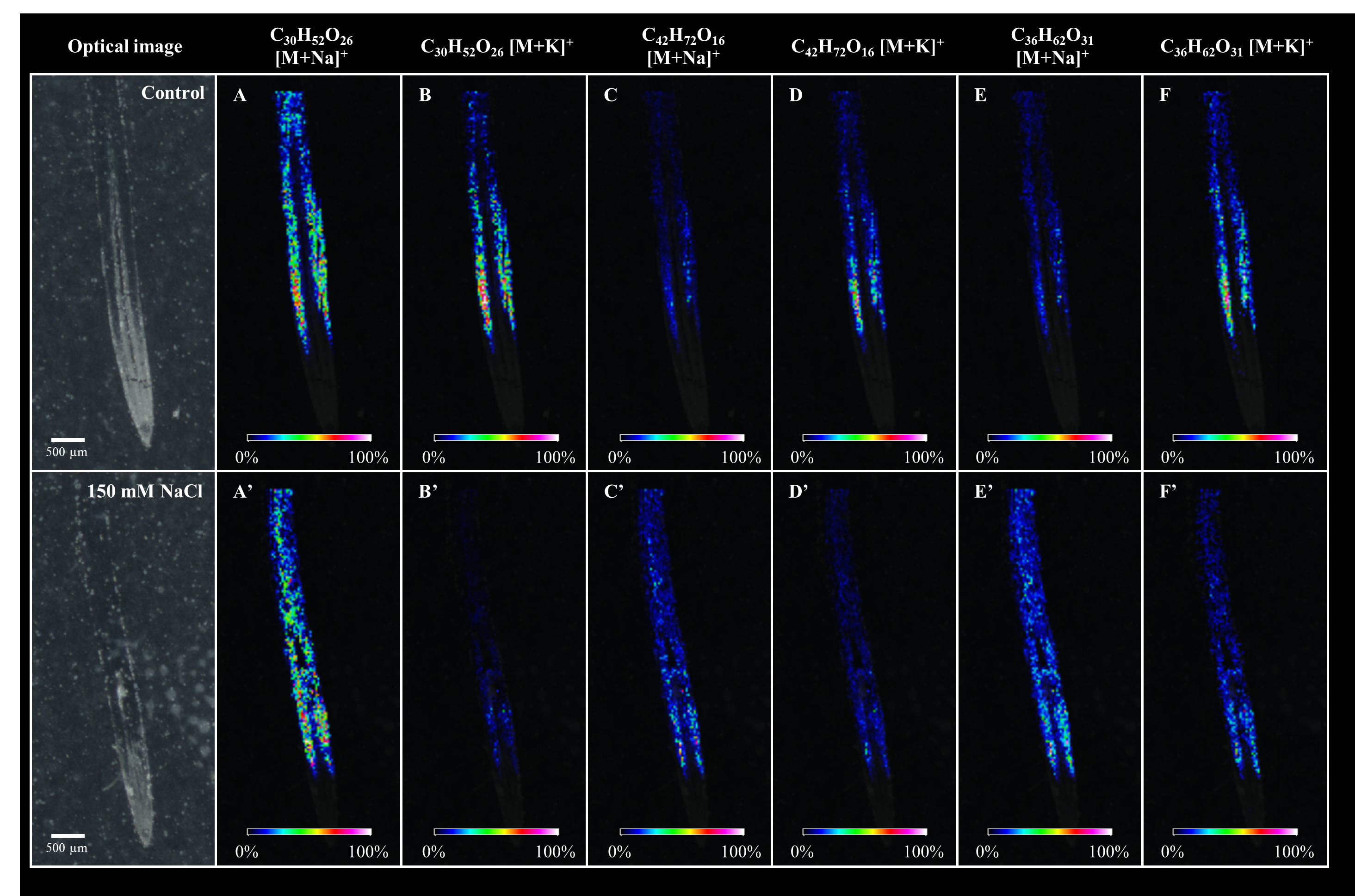

Supplement: Supplementary file 9 — Supplemental Fig. S9. Reconstructed ion images of selected oligosaccharides found in the barley cv. Hindmarsh root, recorded with a scanning step size of 30 μm by 30 μm. Optical image of a longitudinal barley root section grown in control (a) and saline (b) conditions. The ion images are of: A and A’, C30H52O26 [M+Na]+ (m/z 851.2671); B and B’, C30H52O26 [M+K]+ (m/z 867.2444); C and C’, C36H62O31 [M+Na]+ (m/z 1013.312); D and D’, C36H62O31 [M+Na]+ (m/z 1029.284); E and E’, C42H72O36 [M+Na]+ (m/z 1175.36); F and F’, C42H72O36 [M+K]+ (m/z 1191.342). Ion image are displayed using the same intensity scale (Rainbow: 0 – 100). The mass accuracy was at < 5 ppm. Scale bars: 500 μm. Control and salt treated images have been set to the same intensity scale and obtained from the same MALDI-MSI experiment. Supplementary material 9 (TIF 3604 KB) [file 11306_2018_1359_MOESM9_ESM.tif]

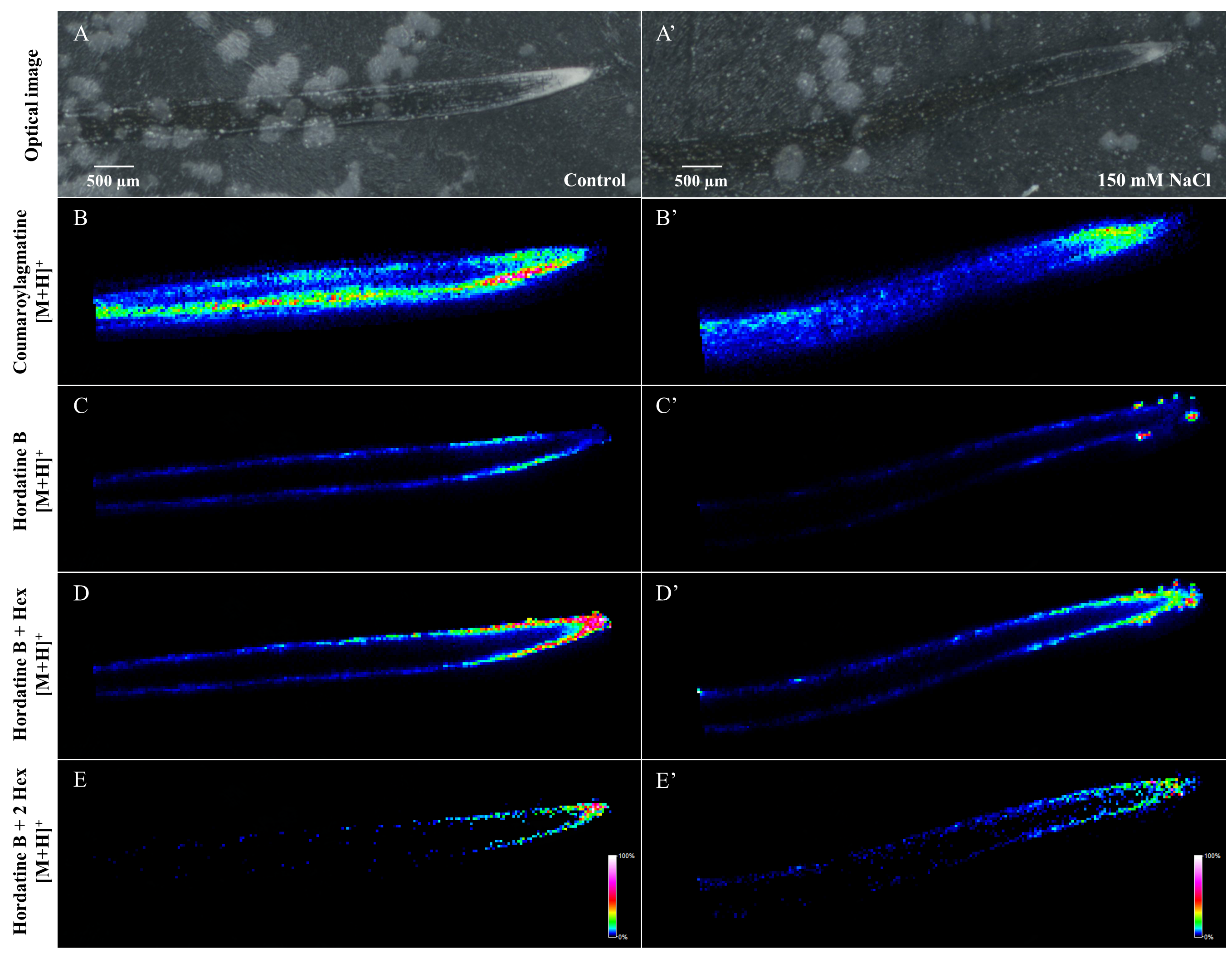

Supplement: Supplementary file 10 — Supplemental Fig. S10. Reconstructed ion images of hydroxycinnamic acid derivatives and hordatines found via MALDI-MSI on barley root sections under control (left panels) and 150 mM NaCl (right panels) conditions. Right panels show a bar graph of the relative concentration of the lipid found on different root zones (Z1 - root cap and cell division zone; Z2 - zone of elongation; Z3 - zone of maturation) under control (C) and salt (S). Images were recorded with a scanning step size of 30 × 30 μm. The MS images are of PC(34:4) (m/z 754.5343), PC(34:3) (m/z 756.5496), PC(34:2) (m/z 758.5674), PC(34:1) (m/z 760.5802), PC(36:6) (m/z 778.5359) and PC(36:5) (m/z 780.5515). Scale bars: 500 μm. Control and salt treated images have been set to the same intensity scale and obtained from the same MALDI-MSI experiment. Supplementary material 10 (TIF 2436 KB) [file 11306_2018_1359_MOESM10_ESM.tif]

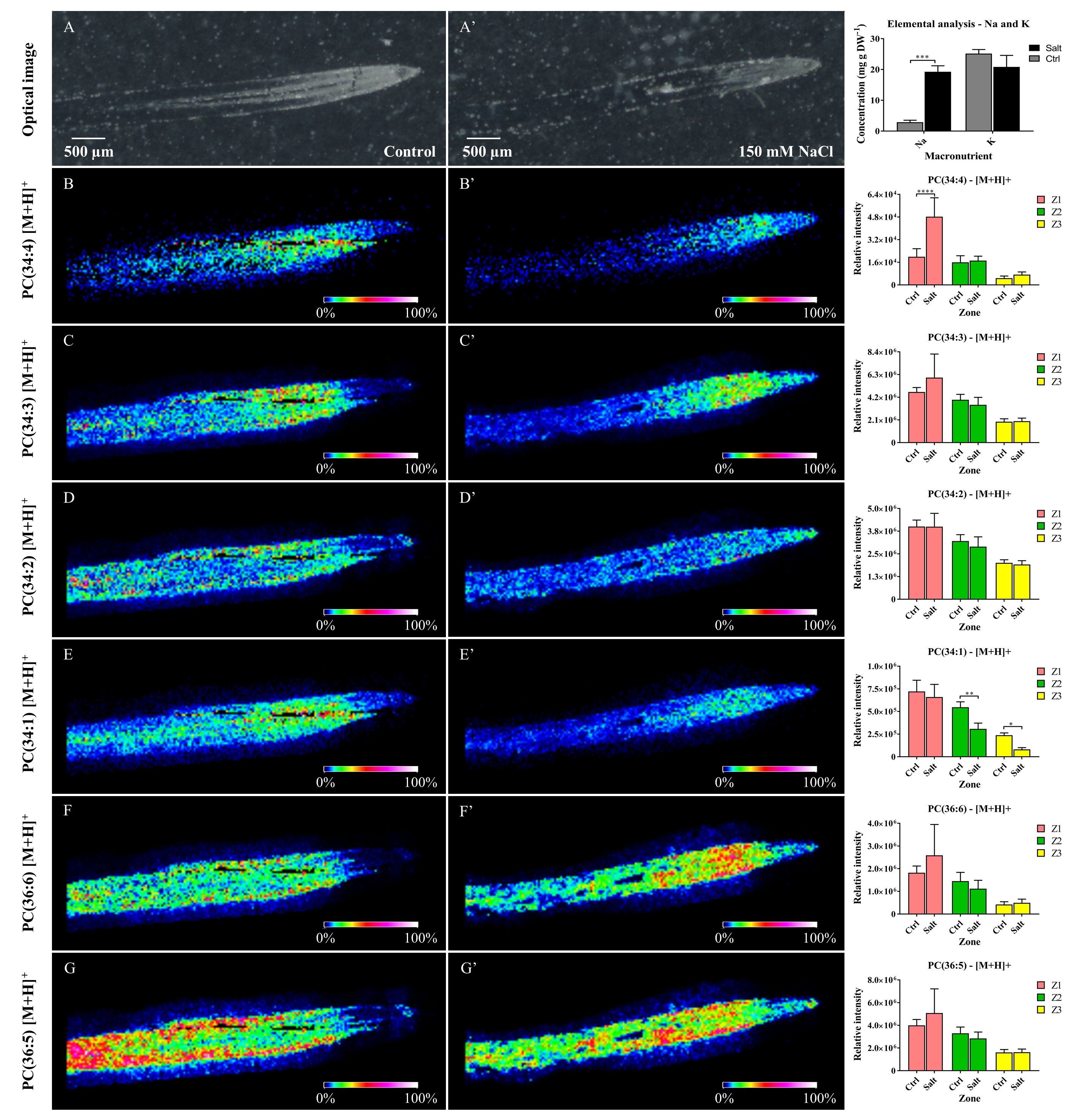

Supplement: Supplementary file 11 — Supplemental Fig. S11. Reconstructed ion images of lipid species found via MALDI-MSI and confirmed by LC-MS on barley root sections under control (left panels) and 150 mM NaCl (middle panels) conditions. Images were recorded with a scanning step size of 30 × 30 μm. The MS images are of p-coumaroylagmatine (m/z 293.1608) as a representative of hydroxycinnamic amides and hordatine B as representative for hordatines: non-glycosylated hordatine B (m/z 581.31892), glycosylated hordatine B (m/z 743.3713) and maltosylated hordatine B (m/z 905.4242). Scale bars: 500 μm. Control and salt treated images have been set to the same intensity scale and obtained from the same MALDI-MSI experiment. Supplementary material 11 (TIF 5089 KB) [file 11306_2018_1359_MOESM11_ESM.tif]

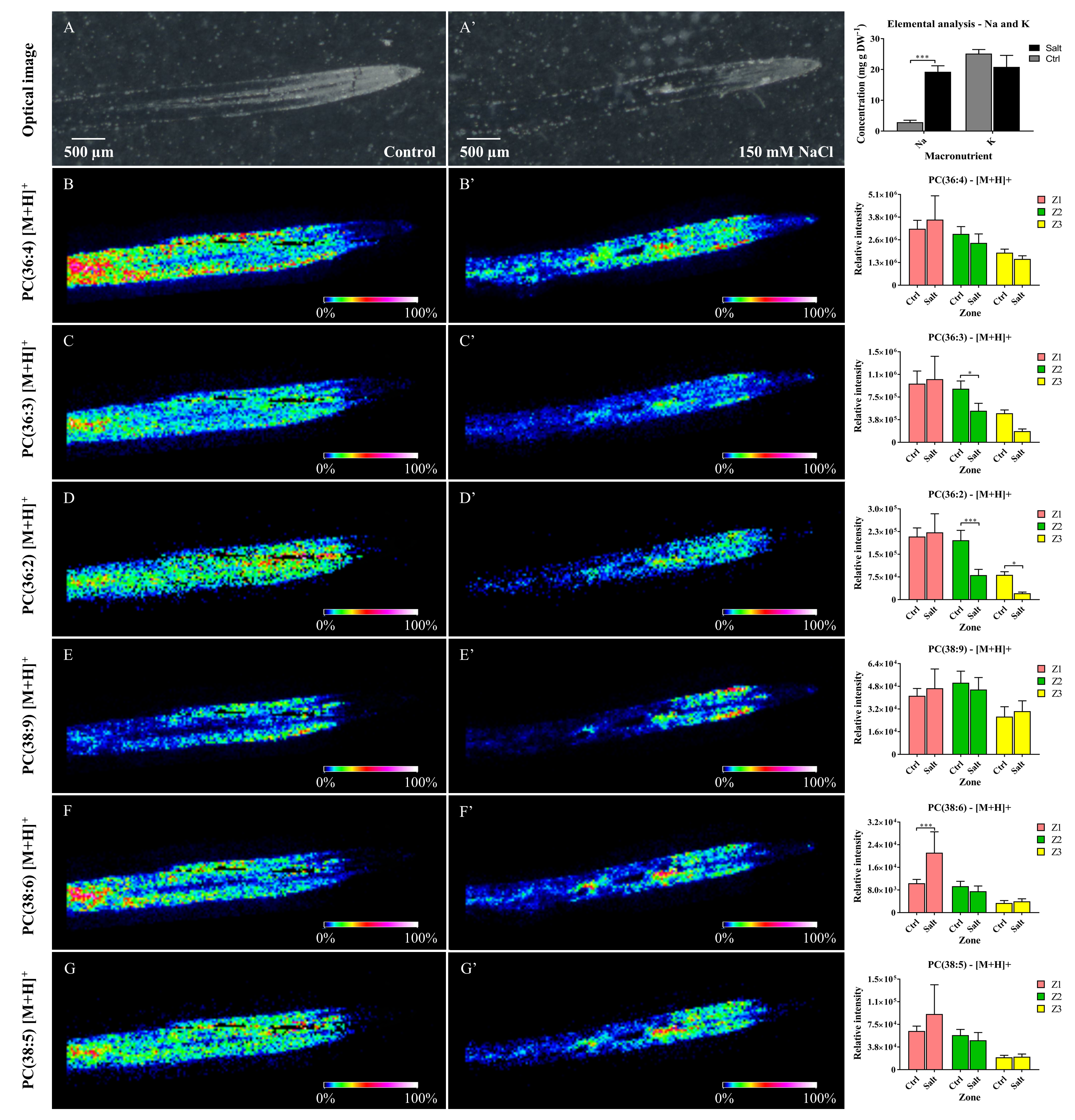

Supplement: Supplementary file 12 — Supplemental Fig. S12. Reconstructed ion images of lipid species found via MALDI-MSI and confirmed by LC-MS on barley root sections under control (left panels) and 150 mM NaCl (middle panels) conditions. Right panels show a bar graph of the relative concentration of the lipid found on different root zones (Z1 - root cap and cell division zone; Z2 - zone of elongation; Z3 - zone of maturation) under control (C) and salt (S). Images were recorded with a scanning step size of 30 × 30 μm. The MS images are of PC(36:4) (m/z 782.57), PC(36:3) (m/z 693.4461), PC(36:2) (m/z 786.6051), PC(38:9) (m/z 800.5252), PC(38:6) (m/z 806.5703) and PC(38:5) (m/z 808.5863). Scale bars: 500 μm. Control and salt treated images have been set to the same intensity scale and obtained from the same MALDI-MSI experiment. Supplementary material 12 (TIF 4427 KB) [file 11306_2018_1359_MOESM12_ESM.tif]

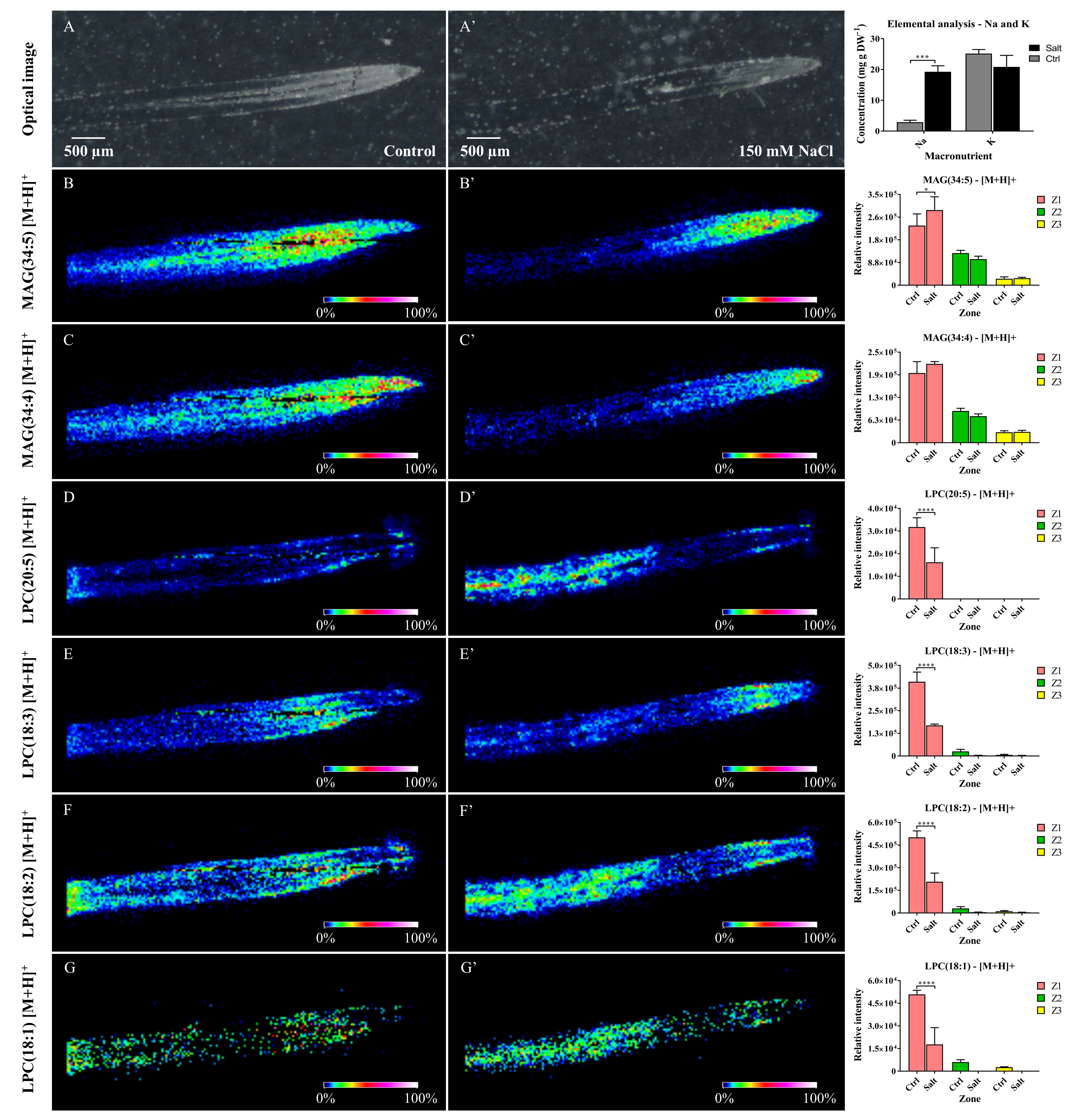

Supplement: Supplementary file 13 — Supplemental Fig. S13. Reconstructed ion images of lipid species found via MALDI-MSI and confirmed by LC-MS on barley root sections under control (left panels) and 150 mM NaCl (middle panels) conditions. Right panels show a bar graph of the relative concentration of the lipid found on different root zones (Z1 - root cap and cell division zone; Z2 - zone of elongation; Z3 - zone of maturation) under control (C) and salt (S). Images were recorded with a scanning step size of 30 × 30 μm. The MS images are of MAG(34:5) (m/z 573.4873), MAG(34:4) (m/z 575.5033), LPC(20:5) (m/z 542.3223), LPC(18:3) (m/z 518.3257), LPC(18:2) (m/z 520.3407) and LPC(18:1) (m/z 522.357). Scale bars: 500 μm. Control and salt treated images have been set to the same intensity scale and obtained from the same MALDI-MSI experiment. Supplementary material 13 (TIF 4225 KB) [file 11306_2018_1359_MOESM13_ESM.tif]

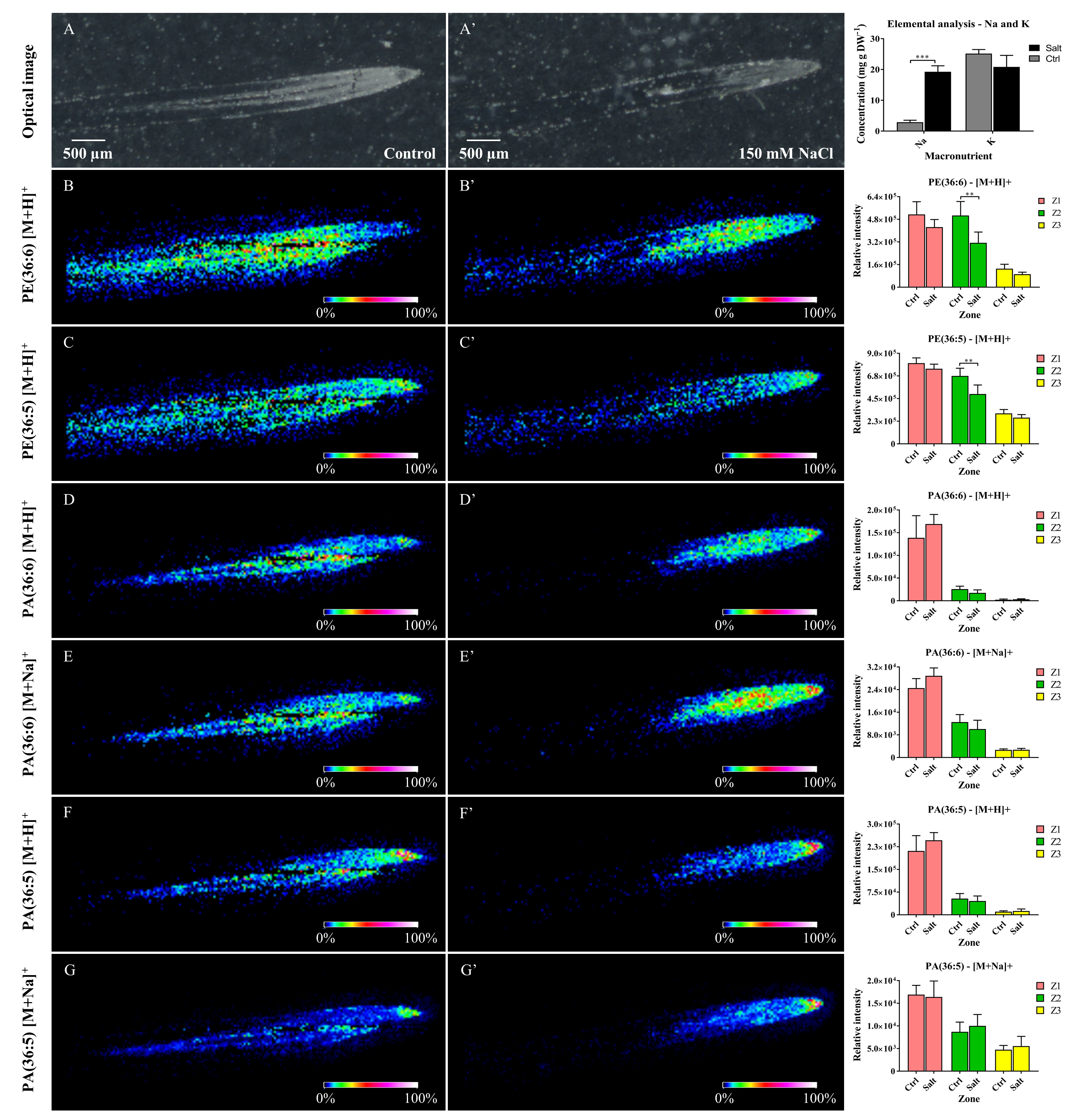

Supplement: Supplementary file 14 — Supplemental Fig. S14. Reconstructed ion images of lipid species found via MALDI-MSI and confirmed by LC-MS on barley root sections under control (left panels) and 150 mM NaCl (middle panels) conditions. Right panels show a bar graph of the relative concentration of the lipid found on different root zones (Z1 - root cap and cell division zone; Z2 - zone of elongation; Z3 - zone of maturation) under control (C) and salt (S). Images were recorded with a scanning step size of 30 × 30 μm. The MS images are of PE(36:6) (m/z 736.4836), PE(36:5) (m/z 738.5002), PA(36:6) (m/z 695.4598), PA(36:6) (m/z 715.4262), PA(36:5) (m/z 693.4461) and PA(36:5) (m/z 717.44232). Scale bars: 500 μm. Control and salt treated images have been set to the same intensity scale and obtained from the same MALDI-MSI experiment.Supplementary material 14 (TIF 4029 KB) [file 11306_2018_1359_MOESM14_ESM.tif]

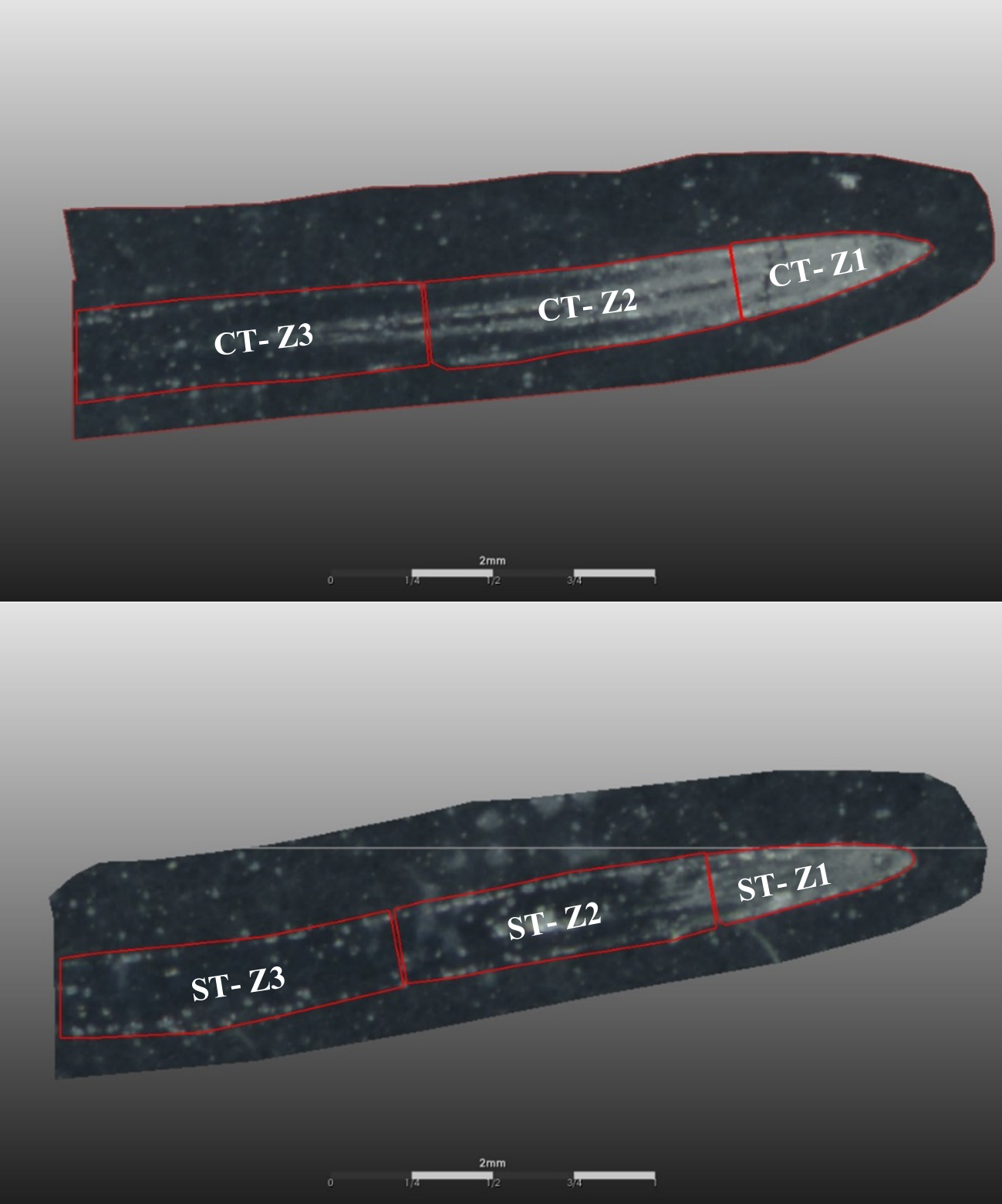

Supplement: Supplementary file 15 — Supplemental Fig. S15. Barley root sections divided by zone for discriminative analysis in SCiLS Lab. CT - control; ST - salt; Z1 - root cap and cell division zone; Z2 - elongation zone; and Z3 - maturation zone. Supplementary material 15 (TIF 798 KB) [file 11306_2018_1359_MOESM15_ESM.tif]
