# Supplementary material for: High-mass-resolution MALDI mass spectrometry imaging reveals detailed spatial distribution of metabolites and lipids in roots of barley seedlings in response to salinity stress
Source: Metabolomics. 2018 Apr 19;14(5):63. doi: 10.1007/s11306-018-1359-3 (PMC5907631; doi:10.1007/s11306-018-1359-3)
Supplement: Supplementary file 16 — Supplementary material 16 (DOCX 18 KB) [file 11306_2018_1359_MOESM16_ESM.docx]

**Supplemental Table S1.** MALDI-MSI identified m/z peaks that showed spatial distribution on longitudinal barley root sections.

| ***m/z*** | ***m/z*** | ***m/z*** | ***m/z*** | ***m/z*** | ***m/z*** | ***m/z*** |
| --- | --- | --- | --- | --- | --- | --- |
| 206.0564 | 312.1465 | 401.2788 | 573.1804 | 753.3838 | 810.5102 | 892.5741 |
| 222.0302 | 315.0710 | 402.2763 | 573.4852 | 754.4760 | 810.6057 | 893.4568 |
| 224.0927 | 315.1444 | 402.2860 | 575.5035 | 754.5343 | 812.5265 | 895.4742 |
| 226.1088 | 316.0790 | 407.2741 | 595.4702 | 755.4016 | 812.6244 | 895.6192 |
| 235.1662 | 318.1354 | 408.2473 | 597.3146 | 756.5149 | 814.4229 | 897.6352 |
| 240.1242 | 320.0910 | 408.2574 | 597.4868 | 756.5496 | 814.4809 | 906.6406 |
| 252.0883 | 320.9765 | 409.2563 | 599.5038 | 757.4199 | 816.4963 | 909.4298 |
| 254.1040 | 322.0342 | 409.2665 | 609.1675 | 758.5308 | 818.5151 | 954.5452 |
| 257.1477 | 322.0564 | 409.2898 | 648.9811 | 758.5674 | 820.5288 | 956.5595 |
| 258.1117 | 323.1359 | 410.2415 | 650.4398 | 759.3665 | 821.4211 | 958.5771 |
| 258.1518 | 331.1131 | 410.2630 | 651.1109 | 759.4304 | 822.5423 | 959.5725 |
| 260.1910 | 331.2010 | 423.2695 | 668.0953 | 760.5804 | 824.5610 | 966.9282 |
| 263.1979 | 347.0874 | 425.2855 | 671.1987 | 761.4466 | 829.6486 | 970.5109 |
| 265.1797 | 347.0968 | 427.3011 | 684.0665 | 771.3787 | 831.6644 | 972.5366 |
| 266.1403 | 357.2511 | 433.1573 | 688.3917 | 772.5471 | 832.5899 | 974.5442 |
| 268.1053 | 358.0465 | 447.1900 | 689.2068 | 774.4460 | 834.5084 | 978.5458 |
| 270.0988 | 361.2119 | 449.1313 | 691.2118 | 776.4621 | 834.6036 | 980.5521 |
| 272.1738 | 361.2355 | 452.0952 | 693.4461 | 776.5196 | 836.5246 | 1013.3120 |
| 273.1216 | 363.0710 | 456.1037 | 695.4598 | 778.5361 | 844.1077 | 1020.1050 |
| 273.1804 | 363.3029 | 462.3620 | 700.0385 | 780.5508 | 848.5599 | 1029.2839 |
| 274.1256 | 365.1060 | 491.0815 | 705.1820 | 782.5463 | 850.5787 | 1036.0739 |
| 275.1207 | 367.0513 | 495.1662 | 709.4167 | 782.5700 | 851.2671 | 1052.0574 |
| 277.1174 | 367.1116 | 496.3429 | 711.4345 | 783.4610 | 853.6519 | 1175.3598 |
| 277.1673 | 376.2994 | 502.3294 | 715.4262 | 784.5900 | 855.5009 | 1191.3411 |
| 280.0928 | 378.2827 | 508.0616 | 717.4423 | 785.1758 | 855.6596 | 1212.0934 |
| 281.2605 | 379.0639 | 509.1495 | 719.4621 | 786.6051 | 857.5183 | 1337.4084 |
| 283.0944 | 379.2827 | 518.3257 | 721.4709 | 789.4703 | 859.6589 | 1353.3827 |
| 283.2762 | 381.0802 | 520.3407 | 731.4002 | 790.4200 | 860.0805 | 1499.4765 |
| 287.1746 | 382.0668 | 522.3570 | 733.4179 | 792.5010 | 861.6717 | 1515.4333 |
| 289.0899 | 382.0850 | 525.1215 | 735.4329 | 794.5122 | 863.6906 | 1661.5133 |
| 291.1469 | 389.2672 | 527.1576 | 736.4863 | 796.5257 | 864.6563 |  |
| 293.1620 | 389.2762 | 530.3464 | 736.5277 | 798.5413 | 866.6714 |  |
| 296.0678 | 390.2838 | 534.2961 | 737.4475 | 800.5252 | 867.2444 |  |
| 298.1429 | 397.1735 | 540.3052 | 738.5002 | 800.5545 | 868.6807 |  |
| 299.0779 | 397.2552 | 542.3223 | 739.4287 | 801.1469 | 871.4767 |  |
| 306.0600 | 397.2647 | 543.1306 | 745.4745 | 802.5400 | 873.4953 |  |
| 306.0826 | 398.0402 | 544.3384 | 747.3730 | 804.5553 | 876.0516 |  |
| 307.1783 | 398.2713 | 556.2799 | 749.3924 | 805.4468 | 880.6327 |  |
| 309.1311 | 399.1473 | 558.2950 | 752.4555 | 806.5703 | 883.6607 |  |
| 310.1304 | 401.2690 | 559.0306 | 752.4997 | 808.5861 | 885.6713 |  |
