# Supplementary material for: High-mass-resolution MALDI mass spectrometry imaging reveals detailed spatial distribution of metabolites and lipids in roots of barley seedlings in response to salinity stress
Source: Metabolomics. 2018 Apr 19;14(5):63. doi: 10.1007/s11306-018-1359-3 (PMC5907631; doi:10.1007/s11306-018-1359-3)
Supplement: Supplementary file 17 — Supplementary material 17 (DOCX 20 KB) [file 11306_2018_1359_MOESM17_ESM.docx]

**Supplemental Table S2.** Maximum AUC-value for the tentatively annotated lipids that were discriminative across different root zones and between control and salt treated roots. WRS, Whole Root Section; Z1, Zone 1; Z2, Zone 2; Z3, Zone 3; AUC -> 0.75 the peak was discriminative in control roots. AUC < 0.25 the peak was discriminative in salt treated roots.

| **Centroid [*m/z*]** | **Name** | **Ion** | **WRS** | **Z1** | **Z2** | **Z3** |
| --- | --- | --- | --- | --- | --- | --- |
| 235.1662 | 1,13-Dihydroxy-herbertene | [M+H]^+^ | **0.141** | **0.033** | **0.074** | **0.19** |
| 277.1174 | (9R,13R)-1a,1b-dihomo-jasmonic acid | [M+K]^+^ | **0.241** | 0.407 | **0.176** | **0.193** |
| 315.0710 | (+)-Bornyl-diphosphate | [M+H]^+^ | **0.042** | 0.015 | **0.013** | **0.074** |
| 331.1131 | 2'-Hydroxymatteucinol | [M+H]^+^ | 0.34 | 0.479 | 0.315 | **0.185** |
| 361.2119 | 12R-HETrE | [M+K]^+^ | 0.386 | 0.278 | **0.242** | 0.546 |
| 361.2355 | 14R,21R-diHDHA | [M+H]^+^ | 0.657 | 0.353 | 0.785 | 0.696 |
| 389.2672 | 1alpha-hydroxy-25,26,27-trinorvitamin D3 24-carboxylic acid | [M+H]^+^ | 0.416 | **0.141** | 0.278 | 0.699 |
| 397.1735 | 4-A4-NeuroP | [M+K]^+^ | **0.13** | **0.175** | **0.062** | **0.105** |
| 397.2552 | PGF2alpha-11-acetate | [M+H]^+^ | **0.758** | 0.34 | **0.821** | **0.898** |
| 398.2713 | S1P(t18:0) | [M+H]^+^ | **0.788** | 0.473 | **0.79** | **0.923** |
| 401.2788 | 24:3(15Z,18Z,21Z) | [M+K]^+^ | 0.595 | 0.448 | 0.469 | **0.761** |
| 409.2563 | 6-deoxyerythronolide B | [M+Na]^+^ | 0.722 | 0.485 | 0.747 | **0.805** |
| 409.2665 | Dioctyl hexanedioate | [M+K]^+^ | 0.707 | 0.485 | 0.717 | **0.8** |
| 410.2630 | CAR(14:0) | [M+K]^+^ | 0.72 | 0.496 | 0.703 | **0.832** |
| 425.2855 | Sorbitan palmitate | [M+Na]^+^ | **0.241** | 0.402 | **0.236** | **0.097** |
| 433.1573 | Prebarbigerone | [M+Na]^+^ | **0.085** | **0.028** | **0.033** | **0.112** |
| 496.3429 | LPC(16:0) | [M+H]^+^ | 0.683 | 0.342 | **0.789** | **0.803** |
| 518.3257 | LPC(18:3) | [M+H]^+^ | 0.507 | 0.239 | 0.712 | 0.413 |
| 530.3464 | HexSph(d20:0) | [M+K]^+^ | **0.109** | **0.218** | **0.052** | **0.093** |
| 542.3223 | LPC(20:5) | [M+H]^+^ | 0.312 | 0.406 | 0.451 | **0.114** |
| 544.3384 | LPC(20:4) | [M+H]^+^ | 0.324 | 0.443 | 0.365 | **0.19** |
| 573.4852 | MAG(34:5) | [M+H]^+^ | 0.715 | 0.359 | **0.817** | **0.902** |
| 575.5035 | MAG(34:4) | [M+H]^+^ | **0.766** | 0.714 | **0.845** | **0.892** |
| 650.4398 | LPS(27:1) | [M+H]^+^ | 0.686 | 0.332 | 0.697 | **0.849** |
| 709.4167 | SQDG(26:1) | [M+H]^+^ | 0.629 | 0.531 | **0.769** | 0.597 |
| 715.4262 | PA(36:6) | [M+Na]^+^ | 0.533 | **0.239** | 0.596 | 0.576 |
| 733.4179 | SQDG(26:0) | [M+Na]^+^ | 0.664 | 0.603 | **0.801** | 0.655 |
| 735.4329 | SQDG(28:2) | [M+H]^+^ | 0.739 | 0.557 | **0.851** | **0.801** |
| 737.4475 | PA(36:3) | [M+K]^+^ | 0.739 | 0.605 | **0.792** | **0.788** |
| 756.5149 | PE(36:4(OH)) | [M+H]^+^ | 0.618 | **0.219** | 0.601 | **0.836** |
| 756.5496 | PC(34:3) | [M+H]^+^ | 0.587 | **0.213** | 0.647 | **0.767** |
| 758.5308 | PE(36:3(OH)) | [M+H]^+^ | 0.665 | 0.301 | 0.602 | **0.872** |
| 760.5804 | PC(34:1) | [M+H]^+^ | 0.687 | 0.423 | **0.75** | **0.814** |
| 778.5361 | PC(36:6) | [M+H]^+^ | 0.442 | **0.223** | 0.311 | 0.634 |
| 782.5463 | SHexCer(d34:0) | [M+H]^+^ | 0.604 | 0.356 | 0.519 | **0.77** |
| 782.5700 | PC(36:4) | [M+H]^+^ | 0.599 | 0.34 | 0.468 | **0.761** |
| 784.5900 | PC(36:3) | [M+H]^+^ | 0.659 | 0.351 | 0.637 | **0.806** |
| 785.1758 | Ptd(S)Ins-(3,4)-P2 (1,2-dioctanoyl) | [M+Na]^+^ | 0.283 | 0.339 | **0.248** | 0.263 |
| 786.6051 | PC(36:2) | [M+H]^+^ | 0.651 | 0.412 | 0.656 | **0.767** |
| 792.5010 | PI-Cer(t32:0) | [M+Na]^+^ | 0.684 | 0.433 | **0.77** | 0.727 |
| 794.5122 | SHexCer(t34:2) | [M+H]^+^ | 0.717 | 0.374 | **0.812** | **0.8** |
| 796.5257 | SHexCer(t34:1) | [M+H]^+^ | 0.732 | 0.48 | **0.802** | **0.799** |
| 798.5413 | SHexCer(t34:0) | [M+H]^+^ | 0.795 | 0.534 | **0.862** | **0.881** |
| 800.5252 | PC(38:9) | [M+H]^+^ | 0.58 | **0.219** | 0.562 | **0.756** |
| 806.5703 | PC(38:6) | [M+H]^+^ | 0.63 | 0.34 | 0.512 | **0.817** |
| 808.5861 | PC(38:5) | [M+H]^+^ | 0.61 | 0.394 | 0.55 | **0.755** |
| 816.4963 | PI-Cer(t34:2) | [M+Na]^+^ | 0.71 | 0.369 | **0.794** | **0.808** |
| 818.5151 | PI-Cer(t34:1) | [M+Na]^+^ | 0.683 | 0.399 | **0.754** | 0.748 |
| 820.5288 | PI-Cer(t34:0) | [M+Na]^+^ | 0.721 | 0.414 | **0.763** | **0.831** |
| 821.4211 | PIP(27:0) | [M+H]^+^ | 0.657 | 0.575 | **0.775** | 0.601 |
| 822.5423 | SHexCer(t36:2) | [M+H]^+^ | **0.758** | 0.428 | **0.8** | **0.882** |
| 824.5610 | PI-Cer(t36:1) | [M+H]^+^ | 0.737 | 0.468 | **0.785** | **0.831** |
| 859.6589 | DAG(51:7) | [M+K]^+^ | 0.664 | 0.35 | 0.739 | **0.761** |
| 861.6717 | DAG(51:6) | [M+K]^+^ | 0.698 | 0.415 | 0.745 | **0.8** |
