# Supplementary material for: High-mass-resolution MALDI mass spectrometry imaging reveals detailed spatial distribution of metabolites and lipids in roots of barley seedlings in response to salinity stress
Source: Metabolomics. 2018 Apr 19;14(5):63. doi: 10.1007/s11306-018-1359-3 (PMC5907631; doi:10.1007/s11306-018-1359-3)
Supplement: Supplementary file 18 — Supplementary material 18 (DOCX 17 KB) [file 11306_2018_1359_MOESM18_ESM.docx]

**Supplemental Table S3.** Maximum AUC-value for the tentatively annotated metabolites that were discriminative across different root zones and between control and salt treated roots. WRS, Whole Root Section; Z1, Zone 1; Z2, Zone 2; Z3, Zone 3; AUC -> 0.75 the peak was discriminative in control roots. AUC < 0.25 the peak was discriminative in salt treated roots.

| **Centroid [*m/z*]** | **Metabolites/Name** | **Ion** | **WRS** | **Z1** | **Z2** | **Z3** |
| --- | --- | --- | --- | --- | --- | --- |
| 258.1117 | Glycerophosphocholine | [M+H]^+^ | **0.107** | **0.023** | **0.018** | **0.122** |
| 224.0927 | Acetyl-L-tyrosine | [M+H]^+^ | **0.167** | **0.051** | **0.152** | **0.205** |
| 226.1088 | 6-Benzylaminopurine | [M+H]^+^ | **0.18** | **0.069** | **0.138** | **0.242** |
| 240.1242 | N-benzyl-1-methyl-1H-pyrazolo [3,4-d]pyrimidin-4-amine | [M+H]^+^ | **0.807** | 0.488 | **0.945** | **0.901** |
| 266.1403 | N(alpha)-Benzyloxycarbonyl-L-leucine | [M+H]^+^ | 0.304 | 0.285 | **0.241** | 0.339 |
| 268.1053 | Adenosine | [M+H]^+^ | 0.65 | 0.397 | 0.658 | **0.785** |
| 316.079 | Queuine | [M+K]^+^ | **0.033** | **0.003** | **0.011** | **0.064** |
| 381.0802 | 2 hexoses | [M+K]^+^ | 0.749 | 0.533 | **0.905** | 0.722 |
| 390.2838 | Sphingofungin B | [M+H]^+^ | 0.487 | **0.213** | 0.268 | 0.734 |
| 398.0402 | Haloxyfop methyl | [M+Na]^+^ | 0.68 | 0.556 | 0.689 | **0.766** |
| 402.286 | Myriocin | [M+H]^+^ | 0.576 | 0.467 | 0.41 | **0.756** |
| 456.1037 | Luteolinidin 3-O-glucoside | [M+Na]^+^ | **0.136** | 0.253 | **0.073** | **0.069** |
| 559.0306 | UDP-D-Xylose | [M+Na]^+^ | 0.354 | 0.499 | 0.322 | **0.249** |
| 573.1804 | Genipin 1-beta-gentiobioside | [M+Na]^+^ | **0.241** | 0.421 | **0.21** | **0.154** |
| 258.1117 | Glycerophosphocholine | [M+H]^+^ | **0.107** | **0.023** | **0.018** | **0.122** |
| 224.0927 | Acetyl-L-tyrosine | [M+H]^+^ | **0.167** | **0.051** | **0.152** | **0.205** |
