# Supplementary material for: High-mass-resolution MALDI mass spectrometry imaging reveals detailed spatial distribution of metabolites and lipids in roots of barley seedlings in response to salinity stress
Source: Metabolomics. 2018 Apr 19;14(5):63. doi: 10.1007/s11306-018-1359-3 (PMC5907631; doi:10.1007/s11306-018-1359-3)
Supplement: Supplementary file 19 — Supplementary material 19 (DOCX 18 KB) [file 11306_2018_1359_MOESM19_ESM.docx]

**Table S4.** Maximum AUC-value for the annotated peaks that were discriminative across different root zones and between control and salt treated roots. WRS, Whole Root Section; Z1, Zone 1; Z2, Zone 2; Z3, Zone 3; AUC -> 0.75 the peak was discriminative in control roots. AUC < 0.25 the peak was discriminative in salt treated roots.

| **Centroid [*m/z*]** | **Unknown/Name** | **Ion** | **WRS** | **Z1** | **Z2** | **Z3** |
| --- | --- | --- | --- | --- | --- | --- |
| 222.0302 | – | – | **0.800** | **0.756** | **0.902** | **0.825** |
| 252.0883 | – | – | **0.098** | **0.035** | **0.070** | **0.118** |
| 254.1040 | – | – | **0.215** | **0.039** | **0.180** | **0.250** |
| 258.1518 | – | – | **0.111** | **0.014** | **0.052** | **0.154** |
| 260.1910 | – | – | 0.494 | **0.183** | 0.276 | 0.743 |
| 265.1797 | – | – | 0.722 | 0.444 | 0.748 | **0.822** |
| 270.0988 | – | – | **0.119** | **0.024** | **0.075** | **0.176** |
| 274.1256 | – | – | 0.290 | **0.197** | **0.182** | 0.383 |
| 275.1207 | – | – | 0.326 | 0.263 | **0.210** | 0.420 |
| 277.1673 | – | – | 0.292 | 0.304 | **0.115** | 0.427 |
| 281.2605 | – | – | **0.155** | **0.051** | **0.113** | **0.236** |
| 283.2762 | – | – | **0.041** | **0.002** | **0.018** | **0.082** |
| 287.1746 | – | – | **0.767** | 0.530 | **0.833** | **0.831** |
| 293.1620 | – | – | **0.203** | 0.310 | **0.138** | **0.131** |
| 310.1304 | – | – | **0.089** | **0.050** | **0.045** | **0.101** |
| 312.1465 | – | – | 0.272 | **0.207** | **0.220** | 0.316 |
| 320.9765 | – | – | **0.788** | 0.637 | **0.812** | **0.859** |
| 363.0710 | – | – | 0.697 | 0.500 | **0.805** | 0.714 |
| 363.3029 | – | – | 0.749 | 0.503 | **0.772** | **0.839** |
| 367.0513 | – | – | 0.276 | 0.499 | 0.265 | **0.130** |
| 376.2994 | – | – | **0.864** | **0.760** | **0.886** | **0.892** |
| 379.0639 | – | – | 0.420 | 0.510 | 0.491 | **0.244** |
| 382.0850 | – | – | 0.731 | 0.525 | **0.880** | 0.707 |
| 389.2762 | – | – | 0.387 | **0.172** | **0.211** | 0.671 |
| 397.2647 | – | – | **0.763** | 0.338 | **0.812** | **0.909** |
| 402.2763 | – | – | 0.580 | 0.464 | 0.429 | **0.750** |
| 407.2741 | – | – | **0.191** | 0.383 | **0.172** | **0.065** |
| 409.2898 | – | – | **0.229** | 0.528 | **0.199** | **0.055** |
| 423.2695 | – | – | 0.263 | 0.400 | 0.283 | **0.106** |
| 427.3011 | – | – | 0.300 | 0.528 | 0.311 | **0.132** |
| 491.0815 | – | – | **0.132** | **0.203** | **0.091** | **0.118** |
| 508.0616 | – | – | **0.178** | **0.034** | **0.117** | 0.268 |
| 599.5038 | – | – | 0.724 | 0.567 | **0.784** | **0.833** |
| 609.1675 | – | – | **0.204** | **0.241** | **0.119** | **0.223** |
| 668.0953 | – | – | **0.044** | **0.034** | **0.032** | **0.059** |
| 684.0665 | – | – | **0.136** | **0.075** | **0.088** | **0.182** |
| 752.4555 | – | – | 0.680 | 0.465 | **0.795** | 0.688 |
| 844.1077 | – | – | **0.192** | **0.233** | **0.186** | **0.176** |
| 860.0805 | – | – | 0.255 | **0.171** | **0.206** | 0.314 |
| 876.0516 | – | – | 0.376 | **0.231** | 0.307 | 0.480 |
