# Supplementary material for: High-mass-resolution MALDI mass spectrometry imaging reveals detailed spatial distribution of metabolites and lipids in roots of barley seedlings in response to salinity stress
Source: Metabolomics. 2018 Apr 19;14(5):63. doi: 10.1007/s11306-018-1359-3 (PMC5907631; doi:10.1007/s11306-018-1359-3)
Supplement: Supplementary file 20 — Supplementary material 20 (DOCX 16 KB) [file 11306_2018_1359_MOESM20_ESM.docx]

**Supplemental Table S5.** Instrumental conditions used for µ-XRF analysis of barley root sections grown under control and high salt (150 mM NaCl) conditions.

| **Acquisition parameters** | | |
| --- | --- | --- |
| Pixel time [ms/pixel] | 20 | |
| Stage speed [mm/s] | 250 | |
|  |  |  |
| **Tube parameter** | | |
| High voltage [kV] | 50 | |
| Anode current [μA] | 200 | |
| Filter | Empty | |
| Optic | Lens | |
| Chamber [mbar] | 20 | |
| Anode | Rh | |
|  |  |  |
| **Detector parameter** | | |
| Selected detectors | 1,2 | |
| Max. pulse throughput [cps] | 130000 | |
