# Supplementary material for: High-mass-resolution MALDI mass spectrometry imaging reveals detailed spatial distribution of metabolites and lipids in roots of barley seedlings in response to salinity stress
Source: Metabolomics. 2018 Apr 19;14(5):63. doi: 10.1007/s11306-018-1359-3 (PMC5907631; doi:10.1007/s11306-018-1359-3)
Supplement: Supplementary file 21 — Supplementary material 21 (DOCX 18 KB) [file 11306_2018_1359_MOESM21_ESM.docx]

**Table S6.** Summary of the number of tentatively annotated lipid species from three root regions of barley cv. Hindmarsh using LC-TripleTOF-MS (positive ionization mode).

| **Lipid class** | **Lipid subclasses** | **Species detected** | **Precursor ion type** |
| --- | --- | --- | --- |
| Fatty acyls (FA) | CAR | 2 | [M+H]^+^ |
|  | FA | 6 | [M+H]^+^ |
|  | WE | 8 | [M+H]^+^, [M+Na]^+^, [M+NH_4_]^+^ |
| Glycerophospholipid (GP) | CL | 2 | [M+H]^+^, [M+NH_4_]^+^ |
|  | PA/LPA | 39/5 | [M+H]^+^, [M+Na]^+^, [M+K]^+^, [M+NH_4_]^+^ |
|  | PC/LPC | 43/9 | [M+H]^+^, [M+K]^+^ |
|  | PE/LPE | 31/2 | [M+H]^+^ |
|  | PG/LPG | 29/6 | [M+H]^+^, [M+Na]^+^, [M+NH_4_]^+^ |
|  | PI/LPI | 12/5 | [M+H]^+^, [M+Na]^+^, [M+K]^+^, [M+NH_4_]^+^ |
|  | PIP | 5 | [M+H]^+^, [M+NH_4_]^+^ |
|  | PS/LPS | 5/4 | [M+H]^+^, [M+K]^+^ |
| Glycerolipids (GL) | MAG | 2 | [M+H]^+^, [M+Na]^+^ |
|  | DAG | 26 | [M+H]^+^, [M+NH_4_]^+^ |
|  | TAG | 35 | [M+H]^+^, [M+Na]^+^, [M+NH_4_]^+^ |
|  | DGDG | 7 | [M+H]^+^, [M+K]^+^, [M+NH_4_]^+^ |
|  | MGDG | 14 | [M+H]^+^, [M+NH_4_]^+^ |
|  | SQDG | 15 | [M+H]^+^, [M+Na]^+^, [M+NH_4_]^+^ |
| Prenol lipids (PR) | Isoprenoid | 5 | [M+H]^+^ |
| Polyketides | Flavonoid | 2 | [M+H]^+^ |
| Sphingolipid (SL) | Cer | 9 | [M+H]^+^, [M+Na]^+^, [M+K]^+^ |
|  | CerP | 6 | [M+H]^+^ |
|  | HexCer | 10 | [M+H]^+^ |
|  | LacCer | 11 | [M+H]^+^ |
|  | LacSph | 1 | [M+H]^+^ |
|  | MIPC | 3 | [M+H]^+^ |
|  | PI-Cer | 6 | [M+H]^+^ |
|  | S1P | 1 | [M+H]^+^ |
|  | SHexCer | 3 | [M+H]^+^ |
|  | Sph | 4 | [M+H]^+^, [M+Na]^+^ |
| Sterol lipids (ST) | CE | 15 | [M+H]^+^, [M+NH_4_]^+^ |
|  | Sulfates | 1 | [M+H]^+^ |

**Abbreviations:** CAR, Acyl Carnitines; CE, Cholesteryl Esters, Cer, Ceramide; CerP, Ceramide phosphate; CL, Cardiolipin; DAG, Diacylglycerol; DGDG, Digalactosyldiacylglycerol; FA, Fatty acyl; HexCer, Hexosylceramides; HexSph, Hexosylsphingosine; LacCer, Lactosylceramide; LacSph, Lactosylsphingosine; LPA, Lysophosphatidic Acid; LPC, Lysophosphatidylcholines; LPIP, Lysophosphatidylinositol Phosphates; LPS, Lipopolysaccharides; MGDG, Monogalactosyldiacylglycerol; MIPC, Mannosyl-inositolphosphoceramides; PA, Phosphatidic Acid; PC, Phosphatidylcholine; PE, Phosphatidylethanolamine; PE-Cer, Phosphoethanolamine-ceramide; PG, Phosphatidylglycerol; PI, Phosphatidylinositol; PI-Cer, Ceramide phosphoinositol; PIP, Phosphoinositide; S1P, Sphingosine-1-phosphate; SHexCer, Sulfatide; Sph, Sphingoid bases; SQDG, Sulfoquinovosyl diacylglycerol, WE, Wax monoester. Pos: positive.
