# Supplementary material for: High-mass-resolution MALDI mass spectrometry imaging reveals detailed spatial distribution of metabolites and lipids in roots of barley seedlings in response to salinity stress
Source: Metabolomics. 2018 Apr 19;14(5):63. doi: 10.1007/s11306-018-1359-3 (PMC5907631; doi:10.1007/s11306-018-1359-3)
Supplement: Supplementary file 22 — Supplementary material 22 (DOCX 15 KB) [file 11306_2018_1359_MOESM22_ESM.docx]

**Supplemental Table S7.** Summary LC-MS results of number of differentially identified lipids as determined by MetaboAnalyst analysis. An absolute log_2_-fold change greater than 2 was required to define a lipid as differentially abundant. Z1, meristematic zone and root cap; Z2, elongation zone; Z3, maturation zone. The full data sets are provided in Supplementary Data Set S1.

|  | | | | **Differentially identified lipids** |
| --- | --- | --- | --- | --- |
| **Treatment-specific** | Control vs Salt | Z1 | Up  Down | 17  33 |
|  |  | Z2 | Up  Down | 61  11 |
|  |  | Z3 | Up  Down | 54  58 |
| **Zone-specific** | Control | Z1 vs Z2 | Up  Down | 180  17 |
|  |  | Z1 vs Z3 | Up  Down | 280  13 |
|  |  | Z2 vs Z3 | Up  Down | 243 |
|  | Salt | Z1 vs Z2 | Up  Down | 248  9 |
|  |  | Z1 vs Z3 | Up  Down | 204  9 |
|  |  | Z2 vs Z3 | Up  Down | 200  4 |
