# Supplementary material for: High-mass-resolution MALDI mass spectrometry imaging reveals detailed spatial distribution of metabolites and lipids in roots of barley seedlings in response to salinity stress
Source: Metabolomics. 2018 Apr 19;14(5):63. doi: 10.1007/s11306-018-1359-3 (PMC5907631; doi:10.1007/s11306-018-1359-3)
Supplement: Supplementary file 23 — Supplementary material 23 (DOCX 34 KB) [file 11306_2018_1359_MOESM23_ESM.docx]

**Table S8.** Tentative LipidMaps (Fahy, Sud et al. 2007) lipid species annotations for all features that showed spatial distribution in barley cv. Hindmarsh root longitudinal sections under control and salt (150 mM NaCl) conditions. Annotations were based on accurate precursor mass search (< 5 ppm).

| ***m/z* [Da]** | **Matched *m/z* [Da]** | **Δ *m/z***  **[mDa]** | **Name** | **Formula** | **Ion** |
| --- | --- | --- | --- | --- | --- |
| 235.1662 | 235.1693 | 3.1 | 1,13-Dihydroxy-herbertene | C_15_H_23_O_2_ | [M+H]^+^ |
| 257.1477 | 257.1512 | 3.5 | 1,13-Dihydroxy-herbertene | C_15_H_22_O_2_Na | [M+Na]^+^ |
| 263.1979 | 263.2006 | 2.7 | 11-Phenylundecanoic acid | C_17_H_27_O_2_ | [M+H]^+^ |
| 265.1797 | 265.1798 | 0.1 | all-trans-7-hydroxyhexadeca-2,4,8,10-tetraenoic acid | C_16_H_25_O_3_ | [M+H]^+^ |
| 273.1216 | 273.1252 | 3.6 | 1,13-Dihydroxy-herbertene | C_15_H_22_O_2_K | [M+K]^+^ |
| 273.1804 | 273.1849 | 4.5 | Beta-estradiol | C_18_H_25_O_2_ | [M+H]^+^ |
| 277.1174 | 277.1201 | 2.7 | (9R,13R)-1a,1b-dihomo-jasmonic acid | C_14_H_22_O_3_K | [M+K]^+^ |
| 283.0944 | 283.0941 | 0.3 | Hemigossypol | C_15_H_16_O_4_Na | [M+Na]^+^ |
| 315.0710 | 315.0711 | 0.0 | (+)-Bornyl-diphosphate | C_10_H_21_O_7_P_2_ | [M+H]^+^ |
| 331.1131 | 331.1176 | 4.5 | 2'-Hydroxymatteucinol | C_18_H_19_O_6_ | [M+H]^+^ |
| 331.2010 | 331.2034 | 2.4 | 3a,17a-Dihydroxy-5b-androstane | C_19_H_32_O_2_K | [M+K]^+^ |
| 361.2119 | 361.2140 | 2.1 | 12R-HETrE | C_20_H_34_O_3_K | [M+K]^+^ |
| 361.2355 | 361.2374 | 1.9 | 14R,21R-diHDHA | C_22_H_33_O_4_ | [M+H]^+^ |
| 367.1116 | 367.1152 | 3.6 | Arjunone | C_19_H_20_O_6_Na | [M+Na]^+^ |
| 379.2827 | 379.2843 | 1.6 | 1-Acetoxy-2-hydroxy-5,12,15-heneicosatrien-4-one | C_23_H_39_O_4_ | [M+H]^+^ |
| 389.2672 | 389.2687 | 1.5 | 1alpha-hydroxy-25,26,27-trinorvitamin D3 24-carboxylic acid | C_24_H_37_O_4_ | [M+H]^+^ |
| 397.1735 | 397.1776 | 4.1 | 4-A4-NeuroP | C_22_H_30_O_4_K | [M+K]^+^ |
| 397.2552 | 397.2585 | 3.3 | PGF2alpha-11-acetate | C_22_H_37_O_6_ | [M+H]^+^ |
| 398.2713 | 398.2666 | 4.7 | S1P(t18:0) | C_18_H_41_NO_6_P | [M+H]^+^ |
| 399.1473 | 399.1439 | 3.4 | 2'-Hydroxypiscerythrinetin | C_22_H_23_O_7_ | [M+H]^+^ |
| 401.2690 | 401.2687 | 0.3 | Ophiobolin A | C_25_H_37_O_4_ | [M+H]^+^ |
| 401.2788 | 401.2817 | 2.9 | 24:3(15Z,18Z,21Z) | C_24_H_42_O_2_K | [M+K]^+^ |
| 408.2473 | 408.2511 | 3.8 | CAR(14:1) | C_21_H_39_NO_4_K | [M+K]^+^ |
| 409.2563 | 409.2560 | 0.3 | 6-deoxyerythronolide B | C_21_H_38_O_6_Na | [M+Na]^+^ |
| 409.2665 | 409.2715 | 5.0 | Dioctyl hexanedioate | C_22_H_42_O_4_K | [M+K]^+^ |
| 410.2630 | 410.2668 | 3.8 | CAR(14:0) | C_21_H_41_NO_4_K | [M+K]^+^ |
| 425.2855 | 425.2873 | 1.8 | Sorbitan palmitate | C_22_H_42_O_6_Na | [M+Na]^+^ |
| 433.1573 | 433.1621 | 4.8 | Prebarbigerone | C_24_H_26_O_6_Na | [M+Na]^+^ |
| 447.1900 | 447.1909 | 0.9 | LPA(16:1) | C_19_H_37_O_7_PK | [M+K]^+^ |
| 449.1313 | 449.1361 | 4.8 | Prebarbigerone | C_24_H_26_O_6_K | [M+K]^+^ |
| 495.1662 | 495.1625 | 3.7 | Lupinisoflavone N | C_25_H_28_O_9_Na | [M+Na]^+^ |
| 496.3429 | 496.3398 | 3.1 | LPC(16:0) | C_24_H_51_NO_7_P | [M+H]^+^ |
| 502.3294 | 502.3268 | 2.6 | HexSph(t18:0) | C_24_H_49_NO_8_Na | [M+Na]^+^ |
| 518.3257 | 518.3241 | 1.6 | LPC(18:3) | C_26_H_49_NO_7_P | [M+H]^+^ |
| 520.3407 | 520.3398 | 0.9 | LPC(18:2) | C_26_H_51_NO_7_P | [M+H]^+^ |
| 522.3570 | 522.3554 | 1.6 | CerP(t26:1) | C_26_H_53_NO_7_P | [M+H]^+^ |
| 530.3464 | 530.3454 | 1.0 | HexSph(d20:0) | C_26_H_53_NO_7_K | [M+K]^+^ |
| 534.2961 | 534.2957 | 0.4 | LPC(16:0) | C_24_H_50_NO_7_PK | [M+K]^+^ |
| 540.3052 | 540.3060 | 0.8 | LPC(18:3) | C_26_H_48_NO_7_PNa | [M+Na]^+^ |
| 542.3223 | 542.3241 | 1.8 | LPC(20:5) | C_28_H_49_NO_7_P | [M+H]^+^ |
| 544.3384 | 544.3398 | 1.4 | LPC(20:4) | C_28_H_51_NO_7_P | [M+H]^+^ |
| 556.2799 | 556.2800 | 0.1 | LPC(18:3) | C_26_H_48_NO_7_PK | [M+K]^+^ |
| 558.2950 | 558.2957 | 0.7 | LPC(18:2) | C_26_H_50_NO_7_PK | [M+K]^+^ |
| 573.4852 | 573.4878 | 2.6 | MAG(34:5) | C_37_H_65_O_4_ | [M+H]^+^ |
| 575.5035 | 575.5034 | 0.1 | MAG(34:4) | C_37_H_67_O_4_ | [M+H]^+^ |
| 595.4702 | 595.4697 | 0.5 | MAG(34:5) | C_37_H_64_O_4_Na | [M+Na]^+^ |
| 597.4868 | 597.4853 | 1.5 | MAG(34:4) | C_37_H_66_O_4_Na | [M+Na]^+^ |
| 650.4398 | 650.4392 | 0.6 | LPS(27:1) | C_33_H_65_NO_9_P | [M+H]^+^ |
| 688.3917 | 688.3951 | 3.3 | LPS(27:1) | C_33_H_64_NO_9_PK | [M+K]^+^ |
| 693.4461 | 693.4490 | 2.9 | PA(36:6) | C_39_H_66_O_8_P | [M+H]^+^ |
| 695.4598 | 695.4647 | 4.8 | PA(36:5) | C_39_H_68_O_8_P | [M+H]^+^ |
| 709.4167 | 709.4192 | 2.5 | SQDG(26:1) | C_35_H_65_O_12_S | [M+H]^+^ |
| 711.4345 | 711.4348 | 0.3 | SQDG(26:0) | C_35_H_67_O_12_S | [M+H]^+^ |
| 715.4262 | 715.4309 | 4.7 | PA(36:6) | C_39_H_65_O_8_PNa | [M+Na]^+^ |
| 717.4423 | 717.4466 | 4.3 | PA(36:5) | C_39_H_67_O_8_PNa | [M+Na]^+^ |
| 719.4621 | 719.4647 | 2.6 | PA(38:7) | C_41_H_68_O_8_P | [M+H]^+^ |
| 731.4002 | 731.4011 | 1.0 | SQDG(26:1) | C_35_H_64_O_12_SNa | [M+Na]^+^ |
| 733.4179 | 733.4167 | 1.2 | SQDG(26:0) | C_35_H_66_O_12_SNa | [M+Na]^+^ |
| 735.4329 | 735.4348 | 1.9 | SQDG(28:2) | C_37_H_67_O_12_S | [M+H]^+^ |
| 736.4863 | 736.4912 | 4.9 | PE(36:6) | C_41_H_71_NO_8_P | [M+H]^+^ |
| 736.5277 | 736.5251 | 2.6 | LPC(34:6) | C_42_H_75_NO_7_P | [M+H]^+^ |
| 737.4475 | 737.4519 | 4.4 | PA(36:3) | C_39_H_71_O_8_PK | [M+K]^+^ |
| 738.5002 | 738.5069 | 6.7 | PE(36:5) | C_41_H_73_NO_8_P | [M+H]^+^ |
| 739.4287 | 739.4309 | 2.3 | PA(38:8) | C_41_H_65_O_8_PNa | [M+Na]^+^ |
| 745.4745 | 745.4779 | 3.4 | PA(38:5) | C_41_H_71_O_8_PNa | [M+Na]^+^ |
| 747.3730 | 747.3751 | 2.1 | SQDG(26:1) | C_35_H_64_O_12_SK | [M+K]^+^ |
| 749.3924 | 749.3907 | 1.7 | SQDG(26:0) | C_35_H_66_O_12_SK | [M+K]^+^ |
| 752.4997 | 752.4977 | 2.1 | SHexCer(d32:1) | C_38_H_74_NO_11_S | [M+H]^+^ |
| 753.3838 | 753.3879 | 4.1 | SQDG(30:7) | C_39_H_61_O_12_S | [M+H]^+^ |
| 754.4760 | 754.4770 | 1.0 | SHexCer(t31:1) | C_37_H_72_NO_12_S | [M+H]^+^ |
| 754.5343 | 754.5382 | 3.9 | PC(34:4) | C_42_H_77_NO_8_P | [M+H]^+^ |
| 755.4016 | 755.4035 | 1.9 | SQDG(30:6) | C_39_H_63_O_12_S | [M+H]^+^ |
| 756.5149 | 756.5174 | 2.5 | PE(36:4(OH)) | C_41_H_75_NO_9_P | [M+H]^+^ |
| 756.5496 | 756.5538 | 4.2 | PC(34:3) | C_42_H_79_NO_8_P | [M+H]^+^ |
| 757.4199 | 757.4192 | 0.7 | SQDG(30:5) | C_39_H_65_O_12_S | [M+H]^+^ |
| 758.5308 | 758.5331 | 2.3 | PE(36:3(OH)) | C_41_H_77_NO_9_P | [M+H]^+^ |
| 758.5674 | 758.5695 | 2.1 | PC(34:2) | C_42_H_81_NO_8_P | [M+H]^+^ |
| 759.4304 | 759.4324 | 2.0 | SQDG(28:1) | C_37_H_68_O_12_SNa | [M+Na]^+^ |
| 760.5804 | 760.5851 | 4.7 | PC(34:1) | C_42_H_83_NO_8_P | [M+H]^+^ |
| 761.4466 | 761.4505 | 3.9 | SQDG(30:3) | C_39_H_69_O_12_S | [M+H]^+^ |
| 771.3787 | 771.3751 | 3.6 | SQDG(28:3) | C_37_H_64_O_12_SK | [M+K]^+^ |
| 772.5471 | 772.5487 | 1.6 | PC(34:3(OH)) | C_42_H_79_NO_9_P | [M+H]^+^ |
| 774.4460 | 774.4471 | 1.1 | PE(36:6) | C_41_H_70_NO_8_PK | [M+K]^+^ |
| 776.4621 | 776.4589 | 3.2 | SHexCer(t31:1) | C_37_H_71_NO_12_SNa | [M+Na]^+^ |
| 776.5196 | 776.5225 | 2.9 | PC(36:7) | C_44_H_75_NO_8_P | [M+H]^+^ |
| 778.5361 | 778.5382 | 2.1 | PC(36:6) | C_44_H_77_NO_8_P | [M+H]^+^ |
| 780.5508 | 780.5538 | 3.0 | PC(36:5) | C_44_H_79_NO_8_P | [M+H]^+^ |
| 782.5463 | 782.5447 | 1.6 | SHexCer(d34:0) | C_40_H_80_NO_11_S | [M+H]^+^ |
| 782.5700 | 782.5695 | 0.5 | PC(36:4) | C_44_H_81_NO_8_P | [M+H]^+^ |
| 783.4610 | 783.4573 | 3.7 | PG(34:3) | C_40_H_73_O_10_PK | [M+K]^+^ |
| 784.5900 | 784.5851 | 4.9 | PC(36:3) | C_44_H_83_NO_8_P | [M+H]^+^ |
| 786.6051 | 786.6008 | 4.4 | PC(36:2) | C_44_H_85_NO_8_P | [M+H]^+^ |
| 789.4703 | 789.4701 | 0.2 | PG(38:9) | C_44_H_70_O_10_P | [M+H]^+^ |
| 792.5010 | 792.4997 | 1.3 | PI-Cer(t32:0) | C_38_H_76_NO_12_PNa | [M+Na]^+^ |
| 794.5122 | 794.5083 | 3.9 | SHexCer(t34:2) | C_40_H_76_NO_12_S | [M+H]^+^ |
| 796.5257 | 796.5240 | 1.8 | SHexCer(t34:1) | C_40_H_78_NO_12_S | [M+H]^+^ |
| 798.5413 | 798.5396 | 1.8 | SHexCer(t34:0) | C_40_H_80_NO_12_S | [M+H]^+^ |
| 800.5252 | 800.5225 | 2.7 | PC(38:9) | C_46_H_75_NO_8_P | [M+H]^+^ |
| 800.5545 | 800.5564 | 1.9 | PC(34:0) | C_42_H_84_NO_8_PK | [M+K]^+^ |
| 802.5400 | 802.5382 | 1.8 | PC(38:8) | C_46_H_77_NO_8_P | [M+H]^+^ |
| 804.5553 | 804.5538 | 1.5 | PC(38:7) | C_46_H_79_NO_8_P | [M+H]^+^ |
| 805.4468 | 805.4498 | 3.0 | PI(31:4(OH)) | C_40_H_70_O_14_P | [M+H]^+^ |
| 806.5703 | 806.5695 | 0.9 | PC(38:6) | C_46_H_81_NO_8_P | [M+H]^+^ |
| 808.5861 | 808.5851 | 1.0 | PC(38:5) | C_46_H_83_NO_8_P | [M+H]^+^ |
| 810.5102 | 810.5069 | 3.3 | PE(42:11) | C_47_H_73_NO_8_P | [M+H]^+^ |
| 810.6057 | 810.6008 | 4.9 | PC(38:4) | C_46_H_85_NO_8_P | [M+H]^+^ |
| 812.5265 | 812.5225 | 4.0 | PE(42:10) | C_47_H_75_NO_8_P | [M+H]^+^ |
| 812.6244 | 812.6294 | 4.9 | CerP(d45:0) | C_45_H_92_NO_6_PK | [M+K]^+^ |
| 814.4229 | 814.4266 | 3.7 | PS(36:8(OH)) | C_42_H_66_NO_11_PNa | [M+Na]^+^ |
| 814.4809 | 814.4784 | 2.5 | PC(36:7) | C_44_H_74_NO_8_PK | [M+K]^+^ |
| 816.4963 | 816.4997 | 3.4 | PI-Cer(t34:2) | C_40_H_76_NO_12_PNa | [M+Na]^+^ |
| 818.5151 | 818.5154 | 0.3 | PI-Cer(t34:1) | C_40_H_78_NO_12_PNa | [M+Na]^+^ |
| 820.5288 | 820.5310 | 2.2 | PI-Cer(t34:0) | C_40_H_80_NO_12_PNa | [M+Na]^+^ |
| 821.4211 | 821.4212 | 0.1 | PIP(27:0) | C_36_H_71_O_16_P_2_ | [M+H]^+^ |
| 822.5423 | 822.5396 | 2.7 | SHexCer(t36:2) | C_42_H_80_NO_12_S | [M+H]^+^ |
| 824.5610 | 824.5648 | 3.8 | PI-Cer(t36:1) | C_42_H_83_NO_12_P | [M+H]^+^ |
| 832.5899 | 832.5851 | 4.8 | PC(40:7) | C_48_H_83_NO_8_P | [M+H]^+^ |
| 834.5084 | 834.5044 | 4.0 | PE(42:10) | C_47_H_74_NO_8_PNa | [M+Na]^+^ |
| 834.6036 | 834.6008 | 2.8 | PC(40:6) | C_48_H_85_NO_8_P | [M+H]^+^ |
| 836.5246 | 836.5225 | 2.2 | PE(44:12) | C_49_H_75_NO_8_P | [M+H]^+^ |
| 848.5599 | 848.5623 | 2.4 | PI-Cer(t36:0) | C_42_H_84_NO_12_PNa | [M+Na]^+^ |
| 850.5787 | 850.5804 | 1.6 | PI-Cer(t38:2) | C_44_H_85_NO_12_P | [M+H]^+^ |
| 855.5009 | 855.5018 | 0.9 | PI(36:6) | C_45_H_76_O_13_P | [M+H]^+^ |
| 857.5183 | 857.5175 | 0.8 | PI(36:5) | C_45_H_78_O_13_P | [M+H]^+^ |
| 859.6589 | 859.6551 | 1.2 | DAG(51:7) | C_54_H_92_O_5_K | [M+K]^+^ |
| 861.6717 | 861.6707 | 1.5 | DAG(51:6) | C_54_H_94_O_5_K | [M+K]^+^ |
| 863.6906 | 863.6864 | 1.6 | DAG(51:5) | C_54_H_96_O_5_K | [M+K]^+^ |
| 864.6563 | 864.6607 | 4.4 | CerP(d49:2) | C_49_H_96_NO_6_PK | [M+K]^+^ |
| 866.6714 | 866.6764 | 5.0 | CerP(d49:1) | C_49_H_98_NO_6_PK | [M+K]^+^ |
| 868.6807 | 868.6790 | 1.7 | PC(42:3) | C_50_H_95_NO_8_P | [M+H]^+^ |
| 871.4767 | 871.4734 | 3.3 | PI(34:3) | C_43_H_77_O_13_PK | [M+K]^+^ |
| 873.4953 | 873.4973 | 2.0 | DGDG(28:1) | C_43_H_78_O_15_K | [M+K]^+^ |
| 880.6327 | 880.6356 | 2.9 | LacCer(t34:0) | C_46_H_90_NO_14_ | [M+H]^+^ |
| 883.6607 | 883.6577 | 3.0 | DAG(53:9) | C_56_H_92_O_5_K | [M+K]^+^ |
| 885.6713 | 885.6710 | 0.3 | DAG(53:8) | C_56_H_94_O_5_K | [M+K]^+^ |
| 892.5741 | 892.5699 | 4.2 | PS(43:7(OH)) | C_49_H_83_NO_11_P | [M+H]^+^ |
| 893.4568 | 893.4576 | 0.8 | LPIP(34:6) | C_43_H_75_O_15_P_2_ | [M+H]^+^ |
| 895.4742 | 895.4733 | 0.9 | LPIP(34:5) | C_43_H_77_O_15_P_2_ | [M+H]^+^ |
| 895.6192 | 895.6187 | 0.5 | PA(49:7) | C_52_H_89_O_8_PNa | [M+Na]^+^ |
| 897.6352 | 897.6368 | 1.6 | PA(51:9) | C_54_H_90_O_8_P | [M+H]^+^ |
| 906.6406 | 906.6430 | 2.4 | PI-Cer(t42:2) | C_48_H_93_NO_12_P | [M+H]^+^ |
| 958.5771 | 958.5723 | 4.8 | PE(50:12) | C_55_H_86_NO_8_PK | [M+K]^+^ |
| 959.5725 | 959.5727 | 0.2 | DGDG(38:9) | C_53_H_83_O_15_ | [M+H]^+^ |
| 980.5521 | 980.5473 | 4.8 | MIPC(d34:1) | C_46_H_88_NO_16_PK | [M+K]^+^ |

Fahy, E., M. Sud, D. Cotter and S. Subramaniam (2007). "LIPID MAPS online tools for lipid research." Nucleic Acids Res **35**(Web Server issue): W606-612.
