# Supplementary material for: High-mass-resolution MALDI mass spectrometry imaging reveals detailed spatial distribution of metabolites and lipids in roots of barley seedlings in response to salinity stress
Source: Metabolomics. 2018 Apr 19;14(5):63. doi: 10.1007/s11306-018-1359-3 (PMC5907631; doi:10.1007/s11306-018-1359-3)
Supplement: Supplementary file 24 — Supplementary material 24 (DOCX 17 KB) [file 11306_2018_1359_MOESM24_ESM.docx]

**Supplemental Table S9.** Summary of the number of tentatively annotated lipid species from longitudinal barley cv. Hindmarsh root sections using MALDI-MSI (positive ionization mode).

| **Lipid class** | **Lipid subclasses** | **Species detected** | **Precursor ion type** |
| --- | --- | --- | --- |
| Fatty acyls (FA) | CAR | 2 | [M+K]^+^ |
|  | FA | 11 | [M+H]^+^, [M+Na]^+^, [M+K]^+^ |
| Glycerophospholipid (GP) | LPS | 1 | [M+H]^+^, [M+K]^+^ |
|  | PA/LPA | 8/1 | [M+H]^+^, [M+Na]^+^, [M+K]^+^ |
|  | PC/LPC | 21/7 | [M+H]^+^, [M+Na]^+^, [M+K]^+^ |
|  | PE | 8 | [M+H]^+^, [M+Na]^+^, [M+K]^+^ |
|  | PG | 2 | [M+H]^+^ |
|  | PI | 4 | [M+H]^+^, [M+K]^+^ |
|  | PIP/LPIP | 1/2 | [M+H]^+^, [M+K]^+^ |
| Glycerolipids (GL) | MAG | 2 | [M+H]^+^, [M+Na]^+^ |
|  | DAG | 5 | [M+H]^+^, [M+Na]^+^, [M+K]^+^ |
|  | DGDG | 2 | [M+H]^+^, [M+K]^+^ |
|  | SQDG | 9 | [M+H]^+^, [M+Na]^+^, [M+K]^+^ |
| Polyketides (PK) | Flavonoids | 5 | [M+H]^+^, [M+Na]^+^, [M+K]^+^ |
| Prenol lipids (PR) | Isoprenoids | 8 | [M+H]^+^, [M+Na]^+^, [M+K]^+^ |
| Sphingolipid (SL) | CerP | 3 | [M+K]^+^ |
|  | HexSph | 2 | [M+K]^+^ |
|  | LacCer | 1 | [M+H]^+^ |
|  | MIPC | 1 | [M+Na]^+^ |
|  | PI-Cer | 8 | [M+H]^+^, [M+Na]^+^ |
|  | S1P | 1 | [M+H]^+^ |
|  | SHexCer | 7 | [M+H]^+^, [M+Na]^+^, [M+K]^+^ |
| Sterol lipids (ST) | Steroids | 2 | [M+H]^+^, [M+K]^+^ |
|  | Secosterol | 1 | [M+H]^+^ |

**Abbreviations:** As provided for Table S6.
