# Supplementary material for: High-mass-resolution MALDI mass spectrometry imaging reveals detailed spatial distribution of metabolites and lipids in roots of barley seedlings in response to salinity stress
Source: Metabolomics. 2018 Apr 19;14(5):63. doi: 10.1007/s11306-018-1359-3 (PMC5907631; doi:10.1007/s11306-018-1359-3)
Supplement: Supplementary file 25 — Supplementary material 25 (DOCX 23 KB) [file 11306_2018_1359_MOESM25_ESM.docx]

**Table S10.** Tentative Metlin (Smith, O'Maille et al. 2005) metabolite annotations for peaks that showed spatial distribution in barley cv. Hindmarsh root longitudinal sections under control and salt (150 mM NaCl) conditions. Annotations were based on accurate precursor mass search (< 5 ppm).

| ***m/z* [Da]** | **Matched *m/z* [Da]** | **Δ *m/z***  **[mDa]** | **Name** | **Formula** | **Ion** |
| --- | --- | --- | --- | --- | --- |
| 224.0927 | 224.0918 | 0.9 | Acetyl-L-tyrosine | C_11_H_13_NO_4_ | [M+H]^+^ |
| 226.1088 | 226.1050 | 3.8 | Acetylcarnitine | C_9_H_17_NO_4_ | [M+Na]^+^ |
| 240.1242 | 240.1244 | 0.2 | N-benzyl-1-methyl-1H-pyrazolo[3,4-d]pyrimidin-4-amine | C_13_H_13_N_5_ | [M+H]^+^ |
| 258.1117 | 258.1101 | 1.6 | Glycerophosphocholine | C_8_H_20_NO_6_P | [M+H]^+^ |
| 268.1053 | 268.1041 | 1.2 | Adenosine | C_10_H_13_N_5_O_4_ | [M+H]^+^ |
| 277.1656 | 277.1659 | 0.3 | p-coumaroylagmatine | C_14_H_20_N_4_O_2_ | [M+H]^+^ |
| 280.0928 | 280.0920 | 0.8 | Glycerophosphocholine | C_8_H_20_NO_6_P | [M+Na]^+^ |
| 293.1608 | 293.1608 | 0.0 | Coumaroyl-hydroxyagmatine | C_14_H_20_N_4_O_3_ | [M+H]^+^ |
| 296.0678 | 296.0660 | 1.8 | Glycerophosphocholine | C_8_H_20_NO_6_P | [M+K]^+^ |
| 306.0600 | 306.0599 | 0.1 | Adenosine | C_10_H_13_N_5_O_4_ | [M+K]^+^ |
| 307.1763 | 307.1765 | 0.2 | Feruloylagmatine | C_15_H_22_N_4_O_3_ | [M+H]^+^ |
| 316.0790 | 316.0840 | 5.0 | Arginyl-Cysteine | C_9_H_19_N_5_O_3_S | [M+K]^+^ |
| 318.1354 | 318.1336 | 1.8 | Piplartine | C_17_H_19_NO_5_ | [M+H]^+^ |
| 322.0564 | 322.0549 | 1.5 | Guanosine | C_10_H_13_N_5_O_5_ | [M+K]^+^ |
| 323.1713 | 323.1714 | 0.1 | Feruloyl-hydroxyagmatine | C_15_H_22_N_4_O_4_ | [M+H]^+^ |
| 382.0668 | 382.0687 | 1.9 | p-Coumaroyl 3-hydroxytyrosine | C_18_H_17_NO_6_ | [M+K]^+^ |
| 390.2838 | 390.285 | 1.2 | Sphingofungin B | C_20_H_39_NO_6_ | [M+H]^+^ |
| 402.2860 | 402.2850 | 1.0 | Sphingofungin F | C_21_H_39_NO_6_ | [M+H]^+^ |
| 408.2574 | 408.2567 | 0.7 | Geranylgeranylcysteine | C_23_H_37_NO_3_S | [M+H]^+^ |
| 456.1037 | 456.1027 | 0.3 | Luteolinidin 3-O-glucoside | C_21_H_21_O_10_ | [M+Na]^+^ |
| 525.1215 | 525.1239 | 2.4 | 5,7,2',3',4'-Pentahydroxy-3,6-dimethoxyflavone 7-glucoside | C_23_H_24_O_14_ | [M+H]^+^ |
| 551.3086 | 551.3089 | 0.3 | Hordatine A | C_28_H_38_N_8_O_4_ | [M+H]^+^ |
| 559.0306 | 559.0337 | 3.1 | UDP-D-Xylose | C_14_H_22_N_2_O_16_P_2_ | [M+Na]^+^ |
| 567.303 | 567.3038 | 0.8 | Hydroxy-hordatine A | C_28_H_38_N_8_O_5_ | [M+H]^+^ |
| 573.1804 | 573.179 | 1.4 | Genipin 1-beta-gentiobioside | C_23_H_34_O_15_ | [M+Na]^+^ |
| 581.3189 | 581.3194 | 0.5 | Hordatine B | C_29_H_40_N_8_O_5_ | [M+H]^+^ |
| 597.3139 | 597.3144 | 0.5 | Hydroxy-hordatine B | C_29_H_40_N_8_O_6_ | [M+H]^+^ |
| 611.3300 | 611.3300 | 0.0 | Hordatine C | C_30_H_42_N_8_O_6_ | [M+H]^+^ |
| 627.3245 | 627.3249 | 0.4 | Hydroxy-hordatine C | C_30_H_42_N_8_O_7_ | [M+H]^+^ |
| 651.1109 | 651.1152 | 4.3 | Glutathione, oxidized | C_20_H_32_N_6_O_12_S_2_ | [M+K]^+^ |
| 713.3611 | 713.3617 | 0.6 | Hordatine A + Hex | C_34_H_48_N_8_O_9_ | [M+H]^+^ |
| 729.3575 | 729.3566 | 0.9 | Hydroxy-hordatine A + Hex | C_34_H_48_N_8_O_10_ | [M+H]^+^ |
| 735.3459 | 735.3436 | 2.3 | Hordatine A + Hex | C_34_H_48_N_8_O_9_ | [M+Na]^+^ |
| 743.3713 | 743.3723 | 1.0 | Hordatine B + Hex | C_35_H_50_N_8_O_10_ | [M+H]^+^ |
| 751.3404 | 751.3385 | 1.9 | Hydroxy-hordatine A + Hex | C_34_H_48_N_8_O_10_ | [M+Na]^+^ |
| 759.3671 | 759.3672 | 0.1 | Hydroxy-hordatine B + Hex | C_35_H_50_N_8_O_11_ | [M+H]^+^ |
| 765.3558 | 765.3542 | 1.6 | Hordatine B + Hex | C_35_H_50_N_8_O_10_ | [M+Na]^+^ |
| 773.3822 | 773.3828 | 0.6 | Hordatine C + Hex | C_36_H_52_N_8_O_11_ | [M+H]^+^ |
| 789.3775 | 789.3777 | 0.2 | Hydroxy-hordatine C + Hex | C_36_H_52_N_8_O_12_ | [M+H]^+^ |
| 801.1469 | 801.1436 | 3.3 | Theogallinin | C_36_H_32_O_21_ | [M+H]^+^ |
| 897.3981 | 897.3965 | 1.6 | Hordatine A + 2 Hex | C_40_H_58_N_8_O_14_ | [M+Na]^+^ |
| 905.4242 | 905.4251 | 0.9 | Hordatine B + 2 Hex | C_41_H_60_N_8_O_15_ | [M+H]^+^ |
| 913.3927 | 913.3914 | 1.3 | Hydroxy-hordatine A + 2 Hex | C_40_H_58_N_8_O_15_ | [M+Na]^+^ |
| 927.4089 | 927.4070 | 1.9 | Hordatine B + 2 Hex | C_41_H_60_N_8_O_15_ | [M+Na]^+^ |
| 943.4032 | 943.4020 | 1.2 | Hydroxy-hordatine B + 2 Hex | C_41_H_60_N_8_O_16_ | [M+K]^+^ |

Smith, C. A., G. O'Maille, E. J. Want, C. Qin, S. A. Trauger, T. R. Brandon, D. E. Custodio, R. Abagyan and G. Siuzdak (2005). "METLIN: a metabolite mass spectral database." Ther Drug Monit **27**(6): 747-751.
