# Supplementary material for: High-mass-resolution MALDI mass spectrometry imaging reveals detailed spatial distribution of metabolites and lipids in roots of barley seedlings in response to salinity stress
Source: Metabolomics. 2018 Apr 19;14(5):63. doi: 10.1007/s11306-018-1359-3 (PMC5907631; doi:10.1007/s11306-018-1359-3)
Supplement: Supplementary file 26 — Supplementary material 26 (DOCX 18 KB) [file 11306_2018_1359_MOESM26_ESM.docx]

**Supplemental Table S11.** Tentative Metlin (Smith, O'Maille et al. 2005) oligosaccharide annotations for peaks that showed spatial distribution in barley cv. Hindmarsh root longitudinal sections under control and salt (150 mM NaCl) conditions. Annotations were based on accurate precursor mass search (< 5 ppm). * Correspond to assignments based on a mass search < 10 ppm.

| ***m/z* [Da]** | **Matched *m/z* [Da]** | **Δ *m/z***  **[mDa]** | **Degree of polymerization** | **Formula** | **Ion** |
| --- | --- | --- | --- | --- | --- |
| 365.1060 | 365.1054 | 0.6 | 2 hexoses | C_12_H_22_O_11_ | [M+Na]^+^ |
| 381.0802 | 381.0794 | 0.8 | 2 hexoses | C_12_H_22_O_11_ | [M+K]^+^ |
| 527.1576 | 527.1583 | 0.7 | 3 hexoses | C_18_H_32_O_16_ | [M+Na]^+^ |
| 543.1306 | 543.1322 | 1.6 | 3 hexoses | C_18_H_32_O_16_ | [M+K]^+^ |
| 689.2068 | 689.2111 | 4.3 | 4 hexoses | C_24_H_42_O_21_ | [M+Na]^+^ |
| 705.1820 | 705.1850 | 3.0 | 4 hexoses | C_24_H_42_O_21_ | [M+K]^+^ |
| 851.2671 | 851.2639 | 3.2 | 5 hexoses | C_30_H_52_O_26_ | [M+Na]^+^ |
| 867.2444 | 867.2378 | 6.6 | 5 hexoses | C_30_H_52_O_26_ | [M+K]^+^ |
| 1013.3120 | 1013.3167 | 4.7 | 6 hexoses | C_36_H_62_O_31_ | [M+Na]^+^ |
| 1029.2839 | 1029.2907 | 6.8* | 6 hexoses | C_36_H_62_O_31_ | [M+K]^+^ |
| 1175.3598 | 1175.3695 | 9.7* | 7 hexoses | C_42_H_72_O*_3_*_6_ | [M+Na]^+^ |
| 1191.3411 | 1191.3435 | 2.4 | 7 hexoses | C_42_H_72_O*_3_*_6_ | [M+K]^+^ |

Smith, C. A., G. O'Maille, E. J. Want, C. Qin, S. A. Trauger, T. R. Brandon, D. E. Custodio, R. Abagyan and G. Siuzdak (2005). "METLIN: a metabolite mass spectral database." Ther Drug Monit **27**(6): 747-751.
